# Quality assessment of flax advanced breeding lines varying in seed coat color and their potential use in the food and industrial applications

**DOI:** 10.1186/s12870-024-04733-1

**Published:** 2024-01-23

**Authors:** Mozhgan Abtahi, Aghafakhr Mirlohi

**Affiliations:** Department of Agronomy and Plant Breeding, College of Agriculture, Isfahan University of Technology, Isfahan, Isfahan, 84156-83111 Iran

**Keywords:** Flax promising lines, Climate change, Metabolic profiling, Lignans, Health benefits, Industrial purposes

## Abstract

**Background:**

With the increasing consumer awareness of the strong relationship between food and health, flax became a promising functional food due to its bioactive nutraceutical composition. Intra-specific crosses of eight contrasting flax genotypes were performed previously, and within segregating F6 progeny families, we investigated a close-up composition of phytochemicals derived from whole seeds.

**Results:**

The considerable genetic variation among the flax F6 families suggested that intra-specific hybridization is essential in flax breeding to obtain and broaden genetic variability and largely affirmed the opportunity for selecting promising lines. Also, significant variations in the targeted metabolite contents and antioxidant properties were observed among brown and yellow-seeded families. Notably, brown-seeded families expressed the highest average values of saturated fatty acids, protein, fiber, tocopherol, phenolics, SDG, and SECO lignans. Yellow-seeded families represented the highest average content of unsaturated fatty acids and mucilage. The cultivation year significantly affects flaxseed’s composition and functional properties, presumably due to temperature, humidity, and sunshine time differences. Interestingly, the seeds obtained in warmer conditions were more potent and had more chemical constituents. The favorable genetic correlations among all evaluated traits suggest the possibility of joint genetic selection for several nutritional and phytochemical characteristics in flax. The current study highlights the importance and utilization of 19 top families as their seeds and oil play imperative roles in the pharmaceuticals and food industries. The antioxidant capacity of the seeds showed that families 84B, 23B, 35Y, 95Y, 30B, 88B, and 78B serve as a natural source of dietary antioxidants beneficial to human health. To increase the oxidative stability of the flaxseed oil, the quality evaluation identified some families with low levels of linolenic acid.

**Conclusions:**

These findings are essential to improving flaxseed’s nutritional quality and therapeutic properties through a bulk breeding program.

**Supplementary Information:**

The online version contains supplementary material available at 10.1186/s12870-024-04733-1.

## Background

Fats and oils are essential constituents of our diet and contribute to the nutritional, textural, and sensory quality of foods. Globally dietary trend is to gradually replace animal fat with vegetable oil to fulfill nutritional requirements in countries where animal fats are consumed at slightly higher levels than vegetable oils [1]. The change is related to the concept of a healthier lifestyle and the need to eat well-balanced food rich in proven beneficial components with a positive influence on health [2]. A Diet rich in saturated fatty acid (SFA) is a suspected contributor to cardiovascular diseases (CVDs) because of an effect to increase in serum low-density lipoprotein (LDL) cholesterol concentrations. In contrast, monounsaturated fatty acids and some polyunsaturated fatty acids (PUFA) from vegetable oil, especially oleic acid, linoleic acid, and linolenic acid, are necessary for overall health by improving neurologic and cardiovascular health and reduction of inflammation [3, 4]. In addition to the type and amount of essential fatty acids, the ideal fatty acid ratio in oilseeds is crucial. The simultaneous consumption of linoleic and linolenic acids in an appropriate ratio (1:1 to 4:1) positively influences the prevention of multiple chronic degenerative disease and cancer [5, 6]. Today, the global oilseed market is dominated by a few crop species, such as soybean, oilseed rape, and sunflower [2, 7]. However, the continuous rise in the human population has led to a surge in demand for high-quality edible oils, which cannot be achieved based on a few major oilseed crops [8]. On the other hand, in sustainable agriculture, the goal is to design low-input farming systems and use alternative crops with regional ecological adaptability, low water demand, economic viability, and high added value. Therefore, it is necessary to increase the production of major oil products, satisfy the markets, and diversify resources by enhancing and expanding the production of newer alternative oil sources [9–11]. Flax (*Linum usitatissimum* L.) has a good position among oilseeds due to its superb nutritional composition and versatile applications. It has become a compelling functional food for extracting valuable oil with good health benefits for humans, as well as for manufacturers’ production (drying oils, paints, textiles, oil cloths or plastics, and printing inks), therapeutic purposes, energy industries, and as roughage for animal nutrition [12–14].

The high protein values of flax make it a preferred dietary source of essential amino acids. Flax is an excellent source of ω-3 fatty acids, especially α-linolenic acid, the richest source of phytoestrogen lignans, such as secoisolariciresinol (SECO) and secoisolariciresinol diglucoside (SDG), a good source of phenols, flavonoids, and gamma-tocopherol (Vitamin E) with appreciable amounts of the water-soluble dietary fiber mucilage, micronutrients such as vitamins (A, B1, E), and minerals (Mg, P, Mn, Se, and Zn) [15, 16]. These valuable phytochemical compounds attribute many beneficial health-promoting properties to flaxseed, such as antioxidant activity, anti-proliferative and anti-inflammatory function, promoting cardiovascular health, and preventing certain types of cancer, hypertension, and diabetes [17]. Also, flax is gluten-free; making it a potential raw material for gluten-free products enriched with proteins and minerals [18].

A vital quality determinant and an essential part of consumer preferences for flax is seed color, which determines seeds’ commercial values. Apart from its direct influence on the acceptability and nutritional quality of flaxseed, the contents of some substances, such as oil, protein, fatty acid profile, and essential breeding traits, depend on seed color [19–21]. It is essential to understand these associations because indirect and sometimes undesirable selection can occur if two traits travel together. The genes involved in seed color may even be pleiotropic, meaning a single gene affects two or more distinct phenotypic traits. Therefore, seed color has been confirmed as a phenotypic marker to be an effective tool for selecting plants with desirable traits [21].

Generally, flax breeding is a more complex undertaking than cereals or legumes because of its multipurpose applications, which require the simultaneous manipulation of quality and agronomic traits [22, 23]. Thus, new flax cultivars must be bred for specific end uses to broaden the use of flaxseed in the food and feed industry. However, breeding new varieties of flax is limited by a need for sufficient variability of the most critical functional traits [24]. As flax is a self-pollinating crop, developing such cultivars involves generating novel genetic variation via manipulating existing genetic resources (e.g., using wide crosses), and then selecting the best transgressive segregants based on end-use quality traits [25]. Moreover, most phytochemicals, antioxidant profiles, and fatty acid composition of flaxseed depend upon the combined activity of several genes, environmental conditions, and crop management [13, 26, 27]. This underscores the significance of characterizing flax germplasm to understand its various biochemical components and to elucidate the fatty acid profiles under climatic factors.

Till now, limited research has targeted nutritional quality improvement in flax, considering oil, protein, and lignans [17, 28]. Therefore, the primary goal of our research was a comprehensive assessment of the amounts of various substances and seed’s biologically active compounds of 100 flax F6 families derived from intra-specific crosses so that promising lines with unique nutritional and phytochemical characteristics can be prioritized for various food, pharmaceutical, and industrial purposes. Evaluating multiple full-sib families, each pair has the same genetic background but differs in seed color, leading to unraveling interrelationships among seed color and nutritional attributes. Furthermore, in light of climate change and the expected increase in the intensity of some extreme weather events, this study intended to best understand the effect of climatic conditions, especially temperature and relative air humidity, on the quality attributes of flax advanced breeding lines.

## Methods

### Generation of flax F6 families

The plant materials for this study were composed of 100 flax families derived from hybridization between 8 linseed genotypes and advanced to F6 generation using the bulk method. The breeding program was started in 2013 at the Isfahan University of Technology, Iran, based on a comprehensive field evaluation of 144 flax genotypes, mainly from the IPK world collection (Gatersleben, Germany), for two years. Based on phenotypic distances among 144 accessions, eight contrasting genotypes were selected due to their contrasting characteristics regarding plant height, number of branches, seed size, fatty acid composition, seed and flower color, which included four yellow-seeded genotypes and four brown-seeded ones (Table 1). Among the selected genotypes, one was an edible-grade oil breeding line (SP1066) with a linolenic acid content of ~ 2.74%. A full-diallel included performing all possible combination crosses among the eight selected parents in two directions (direct and reciprocal crosses) to produce full-sib families of similar genetic backgrounds with different seed colors in a greenhouse during the 2015–2016 growing seasons. The seeds of parental genotypes and their 56 F1 hybrids were grown in a field trial in 2016, and seeds from all plants of each F1 hybrid were collected and bulked to grow F2 families in the next year. The F2 families were advanced to the F3 through the bulk method. In this generation, segregation for seed coat color was observed in some families. The direct and reciprocal crosses of brown-seeded parents produced progenies with only brown seeds (12 families). Crosses of yellow × brown and yellow × yellow parents resulted in yellow and brown-seeded progenies (44 families). Seeds of the F3 generation and parents were subsequently field planted. The yellow and brown-seeded progenies were planted adjacently for families with both seed colors and harvested separately to get 100 F3 entries. F4 seeds were obtained from that generation and advanced by the bulk method to F5 families. These 100 F5 families and eight parental genotypes (108 genotypes) were grown in a field trial in 2020, and seeds from all plants of each F5 family were bulk-harvested to obtain the seeds of F6 families, which were used as genetic materials for this study.

**Table 1:** Information on parental genotypes used to generate F6 segregating families in flax

Parental genotype	code	Origin	Seed color	Flower color
1	Indian	India	Brown	White
2	LTU1474	Lithuania	Brown	White
3	KO37	Iran	Brown	Blue
4	FRA806	France	Brown	Blue
5	FRA771	France	Yellow	White
6	USA1580	USA	Yellow	White
7	SP1066	Canada	Yellow	Blue
8	Golden	Canada	Yellow	Blue

### Assessment of flax F6 families

In 2021, the seeds of F6 families along with their parents were field planted at the Research Farm of Isfahan University of Technology, located in Isfahan, Iran (32°30ʹN, 51°20ʹE), with an average annual temperature and rainfall of 14.5^0^C and 140 mm, respectively. The trials were laid out in an 8 × 8 lattice design with two replications for two consecutive years (2021–2022). Climatic conditions in each cultivation year are shown in Table 2. Each test entry, including the families and parents, was evaluated in single plots of two rows. Each row was 1.5 m long with 0.25 m spacing between the rows and 0.01 m between plants in the row. To investigate the effect of seed coat color on important traits, yellow and brown seeds of each family were planted separately (Table S1). Fertilization and weed control followed standard recommendations for flax throughout the crop growth period.

**Table 2:** Mean monthly minimum and maximum air temperature (Tmin, Tmax), mean monthly relative humidity (RH), mean daily evaporation (Epan) and mean solar radiation (Srad) in 2021 and 2022, no participation occurred during the growing season in both years

	Tmax ^0^C		Tmin ^0^C		RH (%)		Epan (mm/day)		Srad (MJ^− 2^day^− 1^)	
	2021	2022	2021	2022	2021	2022	2021	2022	2021	2022
March	20.40	21.10	6.18	6.87	33.98	32.03	6.14	6.65	18.10	18.60
April	23.30	25.56	8.89	11.70	39.85	36.31	7.24	7.65	20.22	22.70
May	31.11	33.73	15.65	17.43	31.84	27.10	10.50	11.00	25.40	26.30
June	34.19	37.24	19.44	21.51	19.93	15.40	10.80	12.60	28.20	29.50
July	37.28	40.67	21.60	24.21	20.86	16.05	10.90	11.50	27.80	26.90

After full ripening, capsules produced on each family and parent plants were harvested in bulk, thrashed manually, and analyzed for seed quality attributes. Near-infrared (NIR) reflectance spectroscopy analyzer was used to simultaneous detection of quality attributes such as oil content, protein content, fiber content, amino acid profile (aspartic acid, methionine, isoleucine, leucine, lysine, histidine, and arginine), five major fatty acids (palmitic acid, stearic acid, oleic acid, linoleic acid, and linolenic acid), ash content, starch content, and moisture content. For this purpose, a 25-mm sample cup of NIR analyzer was modified by placing a piece of 1.2–1.5 cm thick foam material on the bottom and both insides of the cup to reduce the sample-holding space to a desired size in order that single-plant samples could be analyzed. For each entry, about 10–15 g of cleaned and intact seed was placed separately in a small ring cup and scanned with NIR systems model DA 7250 monochromator instrument (Perten Instruments, Hagersten, Sweden). The cup containing seeds was rotated using a special rotation device attached to the holder during the measurement. This measurement was repeated three times for each sample.

Some parameters provide information about the flax oil nutritional value and heat stability were calculated based on the following Eqs. [29, 30]:

$$\begin{array}{l}{\rm{Omega}}\,{\rm{3}}\,{\rm{to}}\,{\rm{Omega}}\,{\rm{6}}\,{\rm{ratio}}\,\left( {\omega {\rm{3/}}\omega {\rm{6}}} \right)\,{\rm{ = }}\,\\{\rm{\% }}\,{\rm{Linolenic}}\,{\rm{acid}}\,{\rm{/\% Linoleic}}\,{\rm{acid}}\end{array}$$

$$\begin{array}{l}{\rm{Unsaturated}}\,{\rm{to}}\,{\rm{saturated}}\,{\rm{fatty}}\,{\rm{acids}}\,{\rm{ratio}}\,{\rm{(USAT/SAT)}}\,{\rm{ = }}\\{\rm{\% Oleic}}\,{\rm{acid + \% }}\,{\rm{Linoleic}}\,{\rm{acid + \% }}\,{\rm{Linolenic}}\,{\rm{acid/}}\\{\rm{\% Palmitic}}\,{\rm{acid + \% }}\,{\rm{Stearic}}\,{\rm{acid}}\end{array}$$

$$\begin{array}{l}{\rm{Oleic}}\,{\rm{acid}}\,{\rm{to}}\,{\rm{unsaturated}}\,{\rm{fatty}}\,{\rm{acids}}\,{\rm{ratio}}\,\left( {{\rm{OLE/USAT}}} \right)\,{\rm{ = }}\\{\rm{\% Oleic}}\,{\rm{acid/\% }}\,{\rm{Oleic}}\,{\rm{acid + \% }}\,{\rm{Linoleic}}\,{\rm{acid + \% }}\,{\rm{Linolenic}}\,{\rm{acid}}\end{array}$$

The quantitation of the major flaxseed lignans in the extracts obtained from all studied families and parents was performed by high-performance liquid chromatography (HPLC, model Agilent 1090, with diode array detection (DAD) system. On two samples for each family as technical replications, HPLC separation of SDG from flaxseed was performed according to the method described by Mukker et al. [31] with slight modifications. Briefly, 250 mg of seed sample was added to 2.5 ml of the methanol solution (methanol 75%, HPLC grade, Merck) by sonication for 1 h at 45 kHz and 50 °C. The extract was then allowed to cool down at room temperature for at least 30 min and then neutralized (up to pH 7) using acetic acid. The extracts were centrifuged at 5000 rpm for 15 min, and the resultant supernatant was filtered with a 0.22 μm nylon membrane, and each extract’s final volume was made up to 5mL for injection in HPLC. The calibration curve was used for quantifying the lignans, and the results were represented in mg per gram of dry weight. Calibration curves, quantification, and validation of the method were described by Anjum et al. [32].

The seed mucilage content was determined according to the procedure described by Kaewmanee et al. [33] with some modifications. A total of 2 g of seeds were incubated in 20 ml of distilled water at 100 °C for 15 min and stirred with a double-bladed mixer (500 rpm for 24 h) for thermo-stating. Then, the tubes were shaken horizontally at 250 rpm for 30 min. The soluble extract was recovered by relative centrifugal force for 30 min, and the mucilage fraction was precipitated by adding 30 ml of 95% ethanol overnight at 4 °C. Some precipitated impurities were removed by centrifugation; then, the residual ethanol was evaporated at 45 °C for 24 h. The mucilage pellet was weighed and expressed as the ratio between the recovered mucilage’s dry matter content and the seeds’ mass (% w/w).

The total tocopherol content of flax oil was measured using the Folin-Ciocalteu procedure described by Wong et al. [34]. Accordingly, an aliquot of 0.5 mL of flax extract was mixed with 3 ml of distilled water and then added to 2.5 mL Folin-Ciocalteu’s reagent solution (0.2 M). The solution was left to rest for 5 min, and then a 2 mL solution of 7.5% p/v sodium carbonate was added. The resulting blue color was read after 15 min reaction time at 40ºC in a UV-Vis spectrophotometer at 760 nm. The final tocopherol content was calculated by applying a standard curve of pure-tocopherol and expressed as mg of gallic acid equivalent per gram dry mass of oil extract (GAE/g DM).

### Statistical analysis

The mean data of the two replications over the two evaluated years were subjected to descriptive analyses. The combined analysis of variance was carried out using PROC GLM in SAS 9.4 [35] to determine the effects of genotype, year, and genotype × year interaction over each trait. Statistically significant differences among the F6 families and parents means were declared at the probability level of 0.05 by the LSD test. Also, mean comparisons were made between the years to assesse the impact of cultivation year and climatic conditions on the qualitative characteristics of seed and its oil using the LSD test. Statistically significant differences among means of the yellow and brown-seeded families were determined by the LSD test.

According to Falconer and Mackay [36], the phenotypic and genotypic coefficient of variation (PCV and GCV) was computed by the following formula:

1 $${\rm{PCV}}\,{\rm{ = }}\,\left( {{\rm{\sigma p/}}\,{\rm{\mu }}} \right)\,{\rm{ \times }}\,{\rm{100}}$$

2 $${\rm{GCV}}\,{\rm{ = }}\,\left( {{\rm{\sigma g/}}\,{\rm{\mu }}} \right)\,{\rm{ \times }}\,{\rm{100}}$$

Where σp and σg are the phenotypic and genotypic standard deviation, respectively and µ is the phenotypic mean of character.

Broad-sense heritability was estimated using the ratio of Hallauer and Miranda [37]:

3 $${{\rm{h}}^{\rm{2}}}{\rm{b}}\,\left( {{\rm{for}}\,{\rm{combined}}\,{\rm{years}}} \right)\,{\rm{ = }}\,{{\rm{\sigma }}^{\rm{2}}}{\rm{g}}\,{\rm{/}}\,\left( {{{\rm{\sigma }}^{\rm{2}}}{\rm{g}}\,{\rm{ + }}\,{{\rm{\sigma }}^{\rm{2}}}{\rm{gy/y}}\,{\rm{ + }}\,{{\rm{\sigma }}^{\rm{2}}}{\rm{gr/r}}\,{\rm{ + }}{{\rm{\sigma }}^{\rm{2}}}{\rm{e/ry}}} \right)$$

Where σ^2^g is the genotype variance, σ^2^gy is the genotype × year variance, σ^2^gr is the genotype × replication variance, σ^2^e is the environmental variance due to genotype × replication × year interaction, and r and y are the number of replicates and years, respectively.

Principal component analysis (PCA) was performed to reduce the dimensionality of large data sets and also to identify the relationships between traits and dispersion of the F6 families and parents using JMP software (ver. 11.0.0). The genotypic correlations among traits were calculated with two years of phenotypic data in META-R software [38].

## Results

### Effect of genotype on seed quality attributes

All seed quality traits showed significant genotype effects (*P* < 0.01) so that genotype explained a large proportion of the phenotypic variation (50.69–78.24%). The analysis of variance for all quality traits indicated significant differences across F6 families (Table 3). The effect of brown and yellow-seeded families and the parental effects were significant (*p* < 0.01) for all measured traits. The impact of brown versus yellow-seeded families was also substantial for all investigated features (Table 3). On the average of two years, there was a broad genetic difference in protein content ranging from 110.34 g/kg to 288.23 g/kg, showing marked genetic variability among the genotypes (Table 4). The oil content ranged from 20.30 to 53.20%, with a mean value of 46.14% across genotypes. The oil fatty acid profile also varied extensively among genotypes, with an average of 35.99% for linolenic acid (2.74–72.46%), 37.85% for linoleic acid (12.47–61.46%), 25.99% for oleic acid (20.21–32.55%), 12.54% for palmitic acid (9.58–16.65%), and 8.80% for stearic acid (5.24–10.66%) respectively. The extraction yield of mucilage obtained from different flax genotypes varied from 6.75% w/w to 16.20% w/w. The SDG lignan was extended from 9.60 mg/g to 19.76 mg/g with a mean value of 14.15 mg/g. The total phenol and tocopherol contents were 7.70–25.88 mg GAE/g OE and 20.28–55.79 mg/100 g OE, respectively (Table 4).

**Table 3:** Combined ANOVA for important quality traits assessed on 108 flax genotypes (100 F6 families and 8 parental genotypes) during two years

Source of variation	DF	Mean squares																					
		OIL	PRO	FIB	OLE	LIO	LIN	PAL	STR	Omega 3/6	USAT/SAT	OLE/USAT	TTC	TPC	ASP	MET	ISOLEU	LEU	LYS	HIST	SDG	SECO	MUC
Year (Y)	1	60.01^**^	2764.50^**^	205.64^**^	83.49^**^	78.83^**^	41.93^**^	18.94^**^	20.80^**^	26.01^**^	7.88^**^	6.72^**^	30.87^**^	26.25^**^	13.36^**^	11.82^**^	17.32^**^	18.70^**^	9.41^**^	6.34^**^	9.08^**^	10.05^**^	7.95^**^
Genotype (G)	107	228.67^**^	5143.33^**^	398.48^**^	163.96^**^	152.86^**^	76.52^**^	72.80^**^	61.56^**^	67.53^**^	24.18^**^	21.03^**^	89.47^**^	59.14^**^	42.16^**^	36.36^**^	55.17^**^	46.49^**^	26.55^**^	15.47^**^	23.90^**^	26.81^**^	30.03^**^
Parents	7	35.97^**^	2452.36^**^	165.57^**^	65.96^**^	68.41^**^	41.89^**^	27.82^**^	29.17^**^	31.81^**^	11.25^**^	9.87^**^	40.05^**^	37.48^**^	19.77^**^	16.87^**^	28.20^**^	25.57^**^	12.74^**^	7.99^**^	9.23^**^	11.18^**^	14.53^**^
Families	99	21.92^**^	1643.74^**^	76.23^**^	33.04^**^	48.54^**^	16.98^**^	17.89^**^	20.53^**^	26.15^**^	7.77^**^	6.79^**^	27.19^**^	22.23^**^	13.92^**^	11.08^**^	18.79^**^	16.84^**^	10.81^**^	6.58^**^	6.31^**^	7.09^**^	6.95^**^
Yellow (Y)	42	23.17^**^	1789.56^**^	76.03^**^	127.10^**^	46.62^**^	19.02^**^	19.68^**^	31.27^**^	24.68^**^	8.45^**^	8.15^**^	28.54^**^	22.88^**^	14.84^**^	12.29^*^	18.89^**^	18.39^**^	9.59^**^	7.85^**^	4.94^**^	5.62^**^	7.44^**^
Brown (B)	58	23.05^**^	1729.91^**^	75.05^**^	39.97^**^	47.67^**^	28.31^**^	20.54^**^	24.56^**^	28.33^**^	8.14^**^	6.94^**^	28.47^**^	9.96^*^	13.82^**^	11.74^**^	13.98^**^	17.33^**^	7.29^**^	4.26^**^	4.45^**^	6.33^**^	6.96^**^
Y vs. B	1	30.62^ns^	4407.76^**^	302.78^**^	147.64^**^	115.15^**^	78.52^**^	74.25^**^	60.59^**^	69.37^**^	21.36^**^	17.66^**^	76.38^**^	31.96^**^	40.59^**^	17.53^ns^	52.68^**^	47.89	24.80^**^	8.20^ns^	19.77^**^	22.63^**^	30.90^**^
G × Y	107	16.80^**^	634.11^**^	54.78^**^	34.82^**^	30.85^**^	19.96^*^	16.95^**^	14.69^**^	16.36^*^	5.74^**^	4.05^**^	15.87^**^	11.92^**^	8.46^**^	7.43^**^	11.28^**^	10.78^**^	6.24^**^	3.25^**^	4.68^**^	6.04^**^	6.41^**^
Error	214	8.84	662.80	44.75	22.33	17.12	11.32	9.29	7.88	8.03	3.13	2.65	10.56	8.44	5.34	4.50	6.89	5.98	3.45	2.16	2.49	3.05	3.86
TOTAL		292.25	8434.74	619.65	272.76	261.69	140.73	128.98	113.93	131.93	42.93	36.95	154.77	115.75	74.32	64.11	98.66	86.95	45.65	30.52	38.35	44.55	48.25
G		78.24	60.98	64.31	60.11	58.34	54.37	56.44	54.03	51.18	56.32	56.91	57.80	51.09	56.73	56.71	55.92	53.47	58.16	50.69	62.32	60.18	62.24
E		20.27	32.79	33.18	30.61	30.12	29.79	14.68	18.25	19.71	18.35	18.19	19.94	22.68	17.98	18.44	17.55	21.50	20.61	20.77	23.67	22.55	16.47
GE		4.45	7.57	7.64	12.76	11.78	14.18	13.14	12.89	12.40	13.37	10.97	10.25	10.29	11.38	11.58	11.43	12.39	13.66	10.90	12.20	13.55	13.35

^*^P < 0.05, ^**^P < 0.01

OIL, oil content; PRO, protein content; FIB, fiber content; OLE, oleic; LIO, linoleic; LIN, linolenic; PAL, palmitic; STR, stearic; Omega 3/6 linolenic to linoleic ratio; USAT/SAT, unsaturated to saturated ratio; OLE/USAT, oleic to unsaturated ratio; TTC, total tocopherol content; TPC, total phenolic content; ASP, aspartic; MET, methionine; ISOLEU, isoleucine; LEU, leucine; LYS, lysine; HIST, histidine, SDG, secoisolariciresinol diglucoside; SECO, secoisolariciresinol; MUC, mucilage

**Table 4:** Estimates of genetic parameters and phenotypic performance of traits evaluated in 108 flax genotypes (100 F6 families and 8 parental genotypes)

Trait	Mean ± SD	Range	PCV (%)	GCV(%)	H^2^_n_
OIL (%)	46.73 ± 1.03	20.3–53.20	38.03	35.65	0.81
PRO (g/kg)	176.79 ± 15.31	110.34-288.23	36.21	32.14	0.72
FIB (%)	10.70 ± 0.44	7.72–15.22	28.11	24.03	0.63
OLE (%)	25.99 ± 2.15	20.21–32.50	58.44	51.65	0.75
LIO (%)	37.85 ± 2.06	12.47–61.46	60.54	54.51	0.73
LIN (%)	35.99 ± 1.32	2.74–72.46	64.05	57.30	0.74
PAL (%)	12.54 ± 0.88	9.58–16.65	52.10	46.34	0.73
STR (%)	8.80 ± 0.43	5.24–10.66	51.32	46.44	0.70
Omega3/6	1.27 ± 0.01	0.38–18.16	50.44	45.21	0.71
USAT/SAT	5.66 ± 0.03	1.85–9.70	50.87	47.06	0.72
OLE/USAT	0.46 ± 0.01	0.25–0.65	49.34	46.76	0.75
TTC (mg/100 g OE)	36.45 ± 1.99	20.28–55.79	35.47	28.88	0.56
TPC (mg GAE/g OE)	13.85 ± 0.97	7.70-25.88	38.32	25.29	0.60
ASP (%)	1.36 ± 0.02	0.14–4.35	26.42	16.78	0.61
MET (%)	0.07 ± 0.00	0.02–0.31	45.07	32.85	0.65
ISOLE (%)	1.82 ± 0.03	1.25–3.78	39.21	23.14	0.66
LEU (%)	2.58 ± 0.11	1.22–5.23	34.12	28.61	0.62
LYS (%)	1.10 ± 0.01	0.35–1.86	41.14	30.28	0.68
HIS (%)	0.85 ± 0.01	0.25–4.11	35.34	28.70	0.63
SDG (mg/g)	14.15 ± 0.82	9.40-19.76	35.75	29.13	0.68
SECO (mg/g)	6.86 ± 0.05	2.40–17.50	32.09	25.65	0.67
MUC (%)	10.44 ± 0.72	6.75–16.20	32.26	29.73	0.60

OIL, oil content; PRO, protein content; FIB, fiber content; OLE, oleic; LIO, linoleic; LIN, linolenic; PAL, palmitic; STR, stearic; Omega 3/6 linolenic to linoleic ratio; USAT/SAT, unsaturated to saturated ratio; OLE/USAT, oleic to unsaturated ratio; TTC, total tocopherol content; TPC, total phenolic content; ASP, aspartic; MET, methionine; ISOLEU, isoleucine; LEU, leucine; LYS, lysine; HIST, histidine, SDG, secoisolariciresinol diglucoside; SECO, secoisolariciresinol; MUC, mucilage

A considerable variation was recorded for most measured traits, with the genotypic coefficient of variation ranging from 11.67 to 57.41% and the phenotypic variation of 18.03–64.24%. Fatty acid profile, the ratio of linoleic acid to linolenic acid, oleic acid to unsaturated fatty acids ratio, and the ratio of unsaturated to saturated fatty acids had the most considerable genetic variation (45.32–57.41%), while oil and protein content, lignans and mucilage content, tocopherol, phenolics, and essential amino acids had moderately high genetic variation (23.03–35.76%) (Table 4). Heritability estimates were generally high for oil, protein, and fatty acid composition, ranging from 0.70 to 0.81. The estimated values for heritability were relatively high for lignans, mucilage content, and essential amino acids and ranged from 0.60 to 0.69. For tocopherol and phenolics, moderately high heritability of 0.56 and 0.60 was attained (Table 4).

### Effect of environment on seed quality attributes

The average air temperature during the growing season from flowering to maturity stages (the end of May to the end of July) was 21.80^0^ and 24.02^0^ C, and the average daily air temperature reached 24.31^0^ and 28.35^0^ C in 2021 and 2022, respectively (Table 2). In 2022, the average air temperature and pan evaporation were 10.18% and 4.17 mm/day higher than in 2021, meaning a higher crop water requirement in 2022 than in 2021. At this growth stage, the average relative humidity in 2021 (29.30%) was 15.50% higher than in 2022 (25.37%). The results of the analysis of variance revealed substantial differences across cultivation years. Differences among years explained 14.68–33.18% variation for quality traits. The interaction of genotype and year was also significant, accounting for up to 14% of the variation in seed quality attributes (Table 3).

**Fig. 1:**
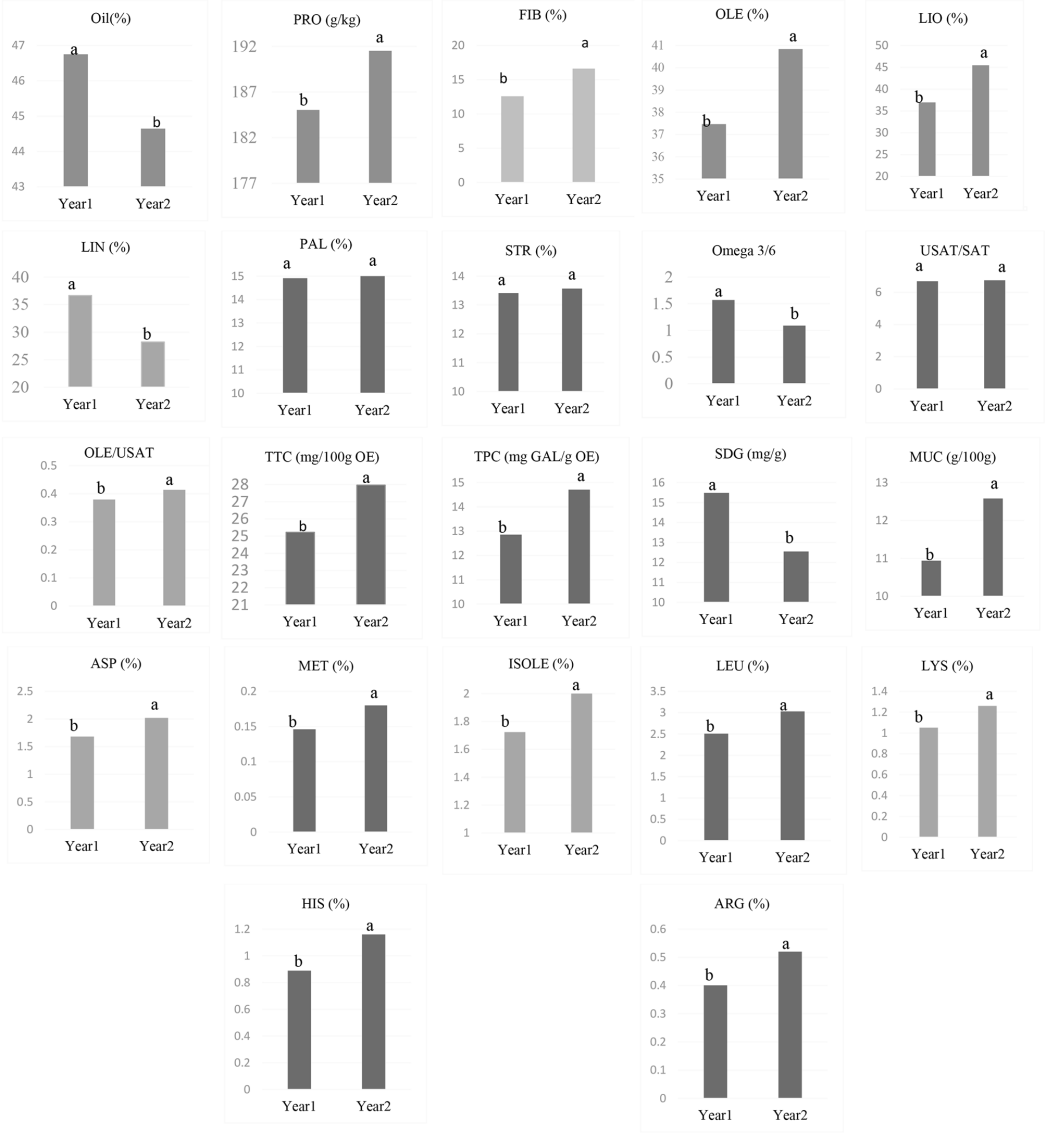
Comparison of two years for measured traits on 108 flax genotypes (100 F6 families and 8 parental genotypes) OIL, oil content; PRO, protein content; FIB, fiber content; OLE, oleic; LIO, linoleic; LIN, linolenic; PAL, palmitic; STR, stearic; Omega 3/6 linolenic to linoleic ratio; USAT/SAT, unsaturated to saturated ratio; OLE/USAT, oleic to unsaturated ratio; TTC, total tocopherol content; TPC, total phenolic content; SDG, secoisolariciresinol diglucoside; SECO, secoisolariciresinol; MUC, mucilage; ASP, aspartic; MET, methionine; ISOLEU, isoleucine; LEU, leucine; LYS, lysine; HIST, histidine, ARG, arginine

The contents of oleic acid, linoleic acid, oleic acid/unsaturated fatty acids, protein, fiber, total phenolic content, tocopherol, mucilage, and essential amino acids in the two years significantly differed, which showed an increasing trend from 2021 to 2022 (Fig. 1). In contrast, the content of oil, linolenic acid, omega3/6, and lignans decreased from the first to the second year. The ratio of unsaturated to saturated fatty acids, palmitic acid, and stearic acid was stable in both experimental years. The variability of ɷ3/ɷ6 in the two years was the highest among the assessed quality attributes, which were 44.03%. The variabilities of SECO lignan, oleic acid, aspartic acid, linolenic acid, SDG lignan, leucine, isoleucine, and arginine remained at a high level (36.75%, 31.96%, 31.94%, 29.87%, 23.44%, 23%, 22.99%, and 20.04%, respectively). In comparison, the variability of lysine and histidine remained low (1.39% and 2.64%) across two years (Fig. 1).

### Relations between seed color and seed quality attributes

**Fig. 2:**
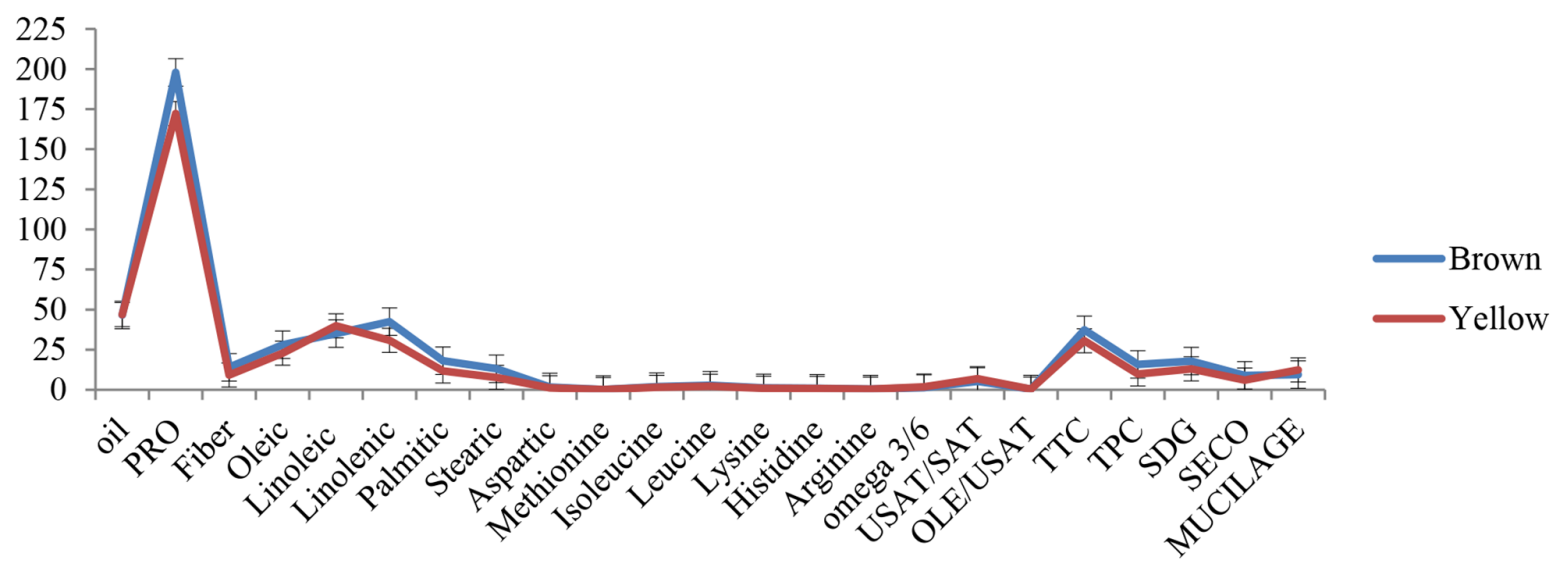
Comparison of mean values for traits evaluated in flax advanced breeding lines and parental genotypes with yellow and brown seed coat colors OIL, oil content; PRO, protein content; FIB, fiber content; OLE, oleic; LIO, linoleic; LIN, linolenic; PAL, palmitic; STR, stearic; Omega 3/6 linolenic to linoleic ratio; USAT/SAT, unsaturated to saturated ratio; OLE/USAT, oleic to unsaturated ratio; TTC, total tocopherol content; TPC, total phenolic content; SDG, secoisolariciresinol diglucoside; SECO, secoisolariciresinol; MUC, mucilage; ASP, aspartic; MET, methionine; ISOLEU, isoleucine; LEU, leucine; LYS, lysine; HIST, histidine, ARG, arginine

To observe the influences of seed color on biologically active compounds, a comparison of multiple full-sib families, each with a common genetic background but different seed colors, was performed (Fig. 2; Table S3). Significant differences were observed among yellow and brown-seeded families within the crosses for all measured traits except oil, methionine, isoleucine, histidine, and arginine. The contents of protein, fiber, tocopherol, phenolics, SDG, and SECO lignans derived from brown-seeded families were 14.91%, 46.76%, 27.78%, 59.95%, 20.28%, and 41.70%, respectively more than the values for yellow-seeded ones. Also, a higher amount of oleic acid (15.07%), palmitic acid (54.35%), stearic acid (49.95%), and oleic acid/unsaturated fatty acids (27.27%) was extracted from brown seed families compared to yellow ones. The average aspartic acid, leucine, and lysine registered higher values of 36.62%, 2.18%, and 22.34% for the brown than yellow-seeded families. On the other hand, yellow-seeded families contained significantly higher values of linoleic acid, linolenic acid, ɷ3/ɷ6 ratio, and unsaturated/saturated fatty acids (10.99%, 37.95%, 45.96%, and 15.13%, respectively) than the brown-seeded families. Comparing seed color regarding mucilage revealed that yellow-seeded families contained higher values (30.69%) than brown-seeded ones (Fig. 2).

### Identification and phenotyping of Flax promising lines

To identify superior flax lines that exhibit contrasting phenotypes regarding nutritional and phytochemical characteristics, a multiple screening analysis was done between 100 F6 families and their parental genotypes (Table S2). In terms of oil, nine brown and nine yellow-seeded families were superior (on average 57% oil content) compared to the other families and parental genotypes with an average of 46.73% oil content. Regarding protein content, eight brown and 11 yellow-seeded families, with an average of 240.45 g/kg were considered superior, while the overall average was 176.79 g/kg. Most families exhibited high fiber content, where six brown and five yellow-seeded families registered the highest values (on average 13.76%) compared with an overall average of 10.70%.

About fatty acid profile, compared to other families and parental genotypes, 12 brown and seven yellow-seeded families were identified as superior for oleic acid content (32.11% on average), six brown and nine yellow-seeded for linoleic acid (with an average of 42.21%), eight brown and 12 yellow-seeded for linolenic acid (with an average of 39.20%), and 13 brown and six yellow-seeded for palmitic and stearic acid contents (with an average of 12.41% and 16.41% respectively). Thirteen brown and eight yellow-seeded families were typically found to contain the lowest levels of linolenic acid (on average 17.94%), compared to the overall average of 35.99%. The seed oil of 11 brown and 11 yellow-seeded families was rich in unsaturated fatty acids with a higher ratio of unsaturated/saturated fatty acids than the overall average. Significantly more essential amino acids were presented in six brown and five yellow-seeded families compared with other genotypes. Regarding lysine content, among 37 brown and nine yellow-seeded families exhibited high values (ranging from 1.2 to 1.87%), 15 families with an average of 1.67% lysine content were notable compared to the overall average of 1%. Thirteen brown and 11 yellow-seeded families were superior for oleic/unsaturated fatty acids (on average 0.44) compared to other families and parental genotypes with an average of 0.36.

Twelve brown and ten yellow-seeded families contained the highest ratio of ɷ3/ɷ6 compared with the other families and parental genotypes. Regarding tocopherol, nine brown and nine yellow-seeded families were superior, with an average of 52.70%, compared with the others having an average of 36.45%. Considering phenolic content, nine brown and nine yellow-seeded families, with an average of 19.89%, were superior to other genotypes, with an overall average of 13.85%. The mean level of SDG and SECO lignans in ten brown and six yellow-seeded families was 10.75% and 21.72% higher than the overall average. Concerning mucilage content, seven brown and seven yellow-seeded families with an average of 11.44% were superior, compared to 10.24%.

### Correlation analysis of seed quality traits

**Fig. 3:**
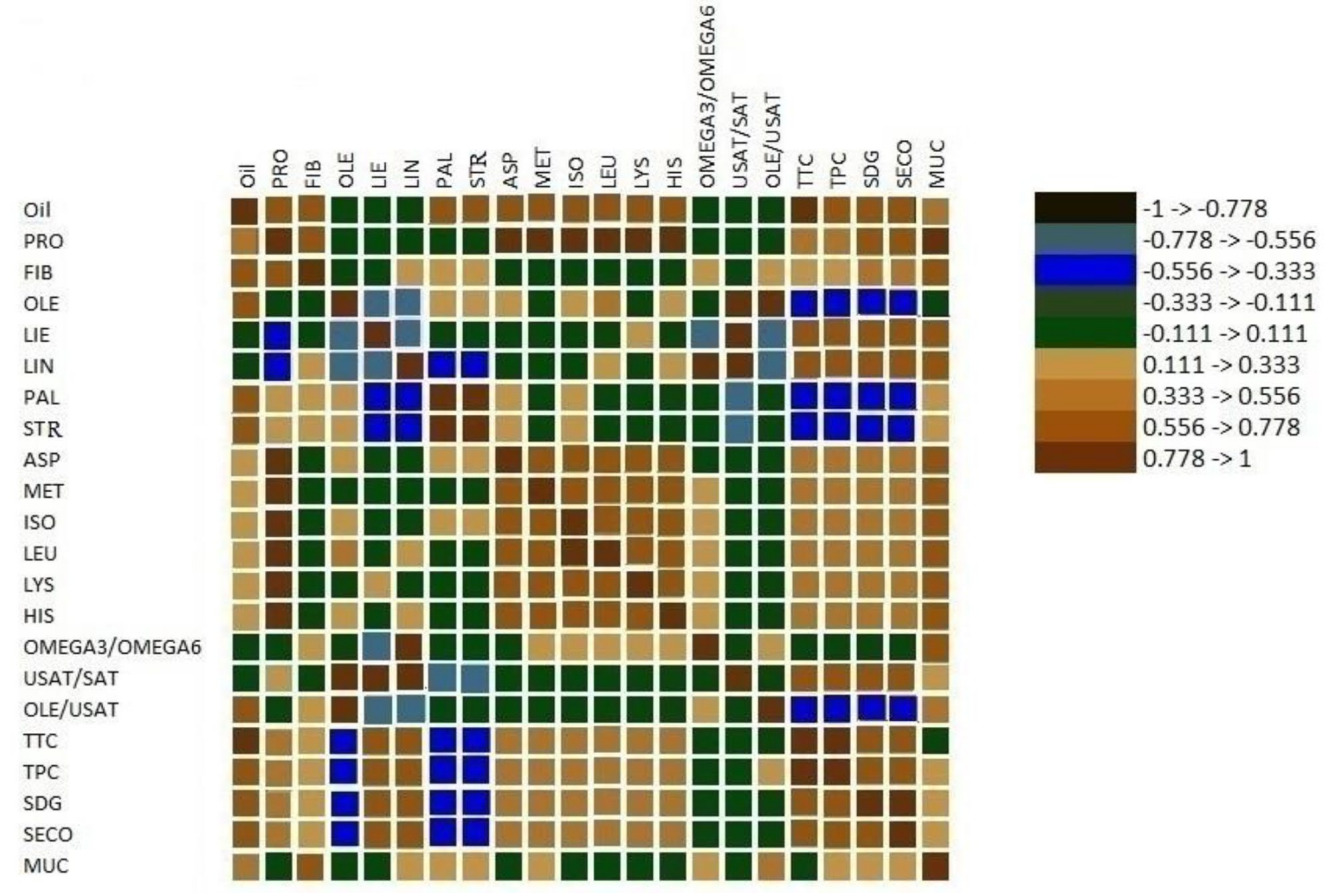
Genetic correlation matrix displaying coefficients among seed oil quality indices with square color indicating direction of effect OIL, oil content; PRO, protein content; FIB, fiber content; OLE, oleic; LIO, linoleic; LIN, linolenic; PAL, palmitic; STR, stearic; Omega 3/6 linolenic to linoleic ratio; USAT/SAT, unsaturated to saturated ratio; OLE/USAT, oleic to unsaturated ratio; TTC, total tocopherol content; TPC, total phenolic content; ASP, aspartic; MET, methionine; ISOLEU, isoleucine; LEU, leucine; LYS, lysine; HIST, histidine, SDG, secoisolariciresinol diglucoside; SECO, secoisolariciresinol; MUC, mucilage

Genotypic correlations among seed compositional traits were investigated by a correlation matrix (Fig. 3; Table S4). Correlation analysis showed that oleic acid was negatively correlated with linoleic acid content (*r* = − 0.75, *p* < 0.01). A negative correlation was also observed between oleic and linolenic acid (*r* = − 0.70, *p* < 0.01), linoleic and palmitic acid (*r* = − 0.53, *p* < 0.01), linoleic and stearic acid (*r* = − 0.51, *p* < 0.01), linolenic and palmitic acid (*r* = − 0.52, *p* < 0.01), linolenic and stearic acid (*r* = − 0.54, *p* < 0.01), and linoleic and linolenic acid (*r* = − 0.70, *p* < 0.01). However, a high positive correlation was found between stearic and palmitic acid (*r* = 0.76, *p* < 0.01). Oil had a positive and almost strong correlation with fiber, tocopherol, phenolics, SDG, and SECO lignans (*r* > 0.65, *p* < 0.01) and a moderate positive association with protein and mucilage (*r* > 0.53, *p* < 0.01). The oil content was positively correlated with the content of oleic acid (*r* = 0.47, *p* < 0.05), palmitic acid (*r* = 0.59, *p* < 0.01), stearic acid (*r* = 0.62, *p* < 0.01), and negatively and insignificantly correlated with the contents of linoleic acid (*r* = -0.30, *p* < 0.1) and linolenic acid (*r* = -0.36, *p* < 0.09). All of the amino acid contents were significantly and positively associated with protein content (*r* > 0.78, *p* < 0.01) and oil content (*r* > 0.64, *p* < 0.01). Meanwhile, all amino acids were strongly correlated with each other (*r* > 0.78, *p* < 0.01).

Protein content, although it had a moderate genetic correlation with fiber, tocopherol, phenolics, SDG, and SECO lignans (*r* > 0.54, *p* < 0.01), was negatively correlated with linolenic and linoleic acid (*r* > 0.55, *p* < 0.01). The results of the correlation analysis showed the presence of some negative correlation between SDG with oleic acid (*r* = -0.53, *p* < 0.01), palmitic acid (*r* = − 0.54, *p* < 0.01), and stearic acid (*r* = -0.51, *p* < 0.01), and positive correlation with tocopherol (*r* = 0.70, *p* < 0.01), and phenolics (*r* = 0.74, *p* < 0.01). A positive correlation was observed for SDG and linoleic acid (*r* = 0.67, *p* < 0.01), linolenic acid (*r* = 0.69, *p* < 0.01), and unsaturated/saturated fatty acids (*r* = 0.71, *p* < 0.01). Tocopherol and phenolics, the two antioxidant indices, showed a robust genetic correlation (*r* = 0.80, *p* < 0.01). Also, correlation analysis suggests a plausible correlation between the two antioxidant indices and linoleic acid, linolenic acid, and unsaturated/saturated fatty acids (*r* > 0.67, *p* < 0.01). Significant positive correlations (*r* > 0.69, *p* < 0.01) were expressed between mucilage and the content of some fatty acids, i.e., linoleic and linolenic, as well as the fiber content and essential amino acids (*r* > 0.54, *p* < 0.01).

### Differentiation of flax genotypes based on biplot analysis

**Fig. 4:**
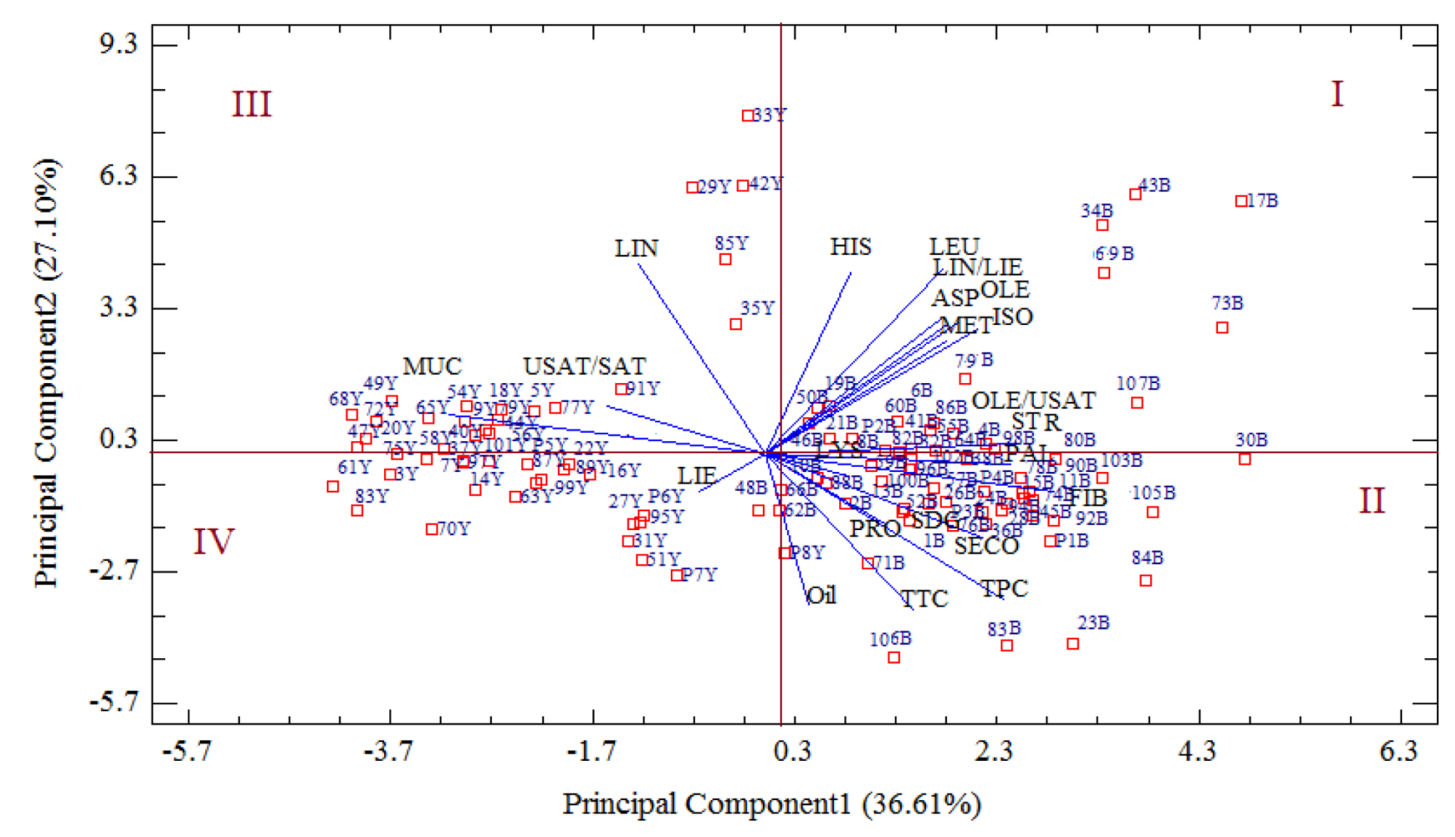
The biplot depicting the scatter of 108 flax genotypes (100 F6 families and 8 parental genotypes) alongside the measured traits in two years of evaluation Oil, oil content; PRO, protein content; FIB, fiber content; OLE, oleic; LIO, linoleic; LIN, linolenic; PAL, palmitic; STR, stearic; Omega 3/6 linolenic to linoleic ratio; USAT/SAT, unsaturated to saturatedratio; OLE/USAT, oleic to unsaturated ratio; TTC, total tocopherol content; TPC, total phenolic content; ASP, aspartic; MET, methionine; ISOLEU, isoleucine; LEU, leucine; LYS, lysine; HIST, histidine, SDG, secoisolariciresinol diglucoside; SECO, secoisolariciresinol; MUC, mucilage

A PCA of the whole data set (108 flax genotypes) was conducted to analyze distributions and associations among the biologically active compounds isolated from the yellow and brown-seeded F6 families. Five principal components (PCs) were obtained, and the related parameters of the first two PCs were calculated. The eigenvalues of PC1 and PC2 explained 63.71% of the total variance (Fig. 4). Fiber content (31.72%), oleic/unsaturated fatty acids (27.98%), palmitic acid (26.92%), stearic acid (26.92%), phenolics (26.15%), SDG (23.95%), SECO (23.95%), and mucilage (-34.57%) made the most considerable contributions to the variance along PC1. Linolenic acid (35.83%), leucine (34.79%), histidine (34.22%), tocopherol (-29.82%), oil (-28.58%), and phenolics (-27.73%) were the major contributors to the PC2. The PCA scatter plot allowed the segregation of F6 families according to the seed coat color. Families with a brown seed color were occupied in the right half of the biplot and had higher levels of oil, protein, fiber, oleic acid, stearic acid, palmitic acid, oleic acid/unsaturated fatty acids, omega3/6, essential amino acids, and lignans. Yellow-seeded families were positioned on the left-hand side of the biplot and had higher levels of unsaturated fatty acids, unsaturated/saturated fatty acids, and mucilage. Based on the positive or negative signs of the sectors of the PC1 and PC2, 100 F6 families were placed in 4 different quartiles of biplot. The group “I” was formed by 24 brown-seeded families on the positive side of both PC1 and PC2 and densely scattered close to the saturated fatty acids, oleic acid/unsaturated fatty acids, and amino acid lysine. Group “II” included 34 brown-seeded families on the positive side of PC1 and the negative side of PC2 and distributed around the oil, protein, lignans, fiber, tocopherol, and phenolics. Twenty-six yellow-seeded families on the negative side of PC1 and positive side of PC2 were more diverse around the mucilage, linolenic acid, and unsaturated/saturated fatty acids and were grouped in region “III”. Finally, sixteen yellow-seeded families were found on the negative side of both PC1 and PC2, scattered toward the linoleic acid and grouped in region IV of the biplot (Fig. 4).

## Discussion

Estimating genetic parameters in segregating populations is an essential activity to direct the breeding program in selecting the most promising genotypes. Marked genetic variation among different F6 families regarding protein content, oil content, fatty acid composition, and other oil quality indicators, including antioxidants and mucilage, can fulfill the purpose of flax breeding in evolving high-quality cultivars (Table 3).

The heritability values for seed quality traits were estimated as relatively high magnitude, ranging from 0.60 to 0.93 (Table 4), indicating low environmental influence on the expression of the traits, considering that this influence prevents the effective selection of genotypes [14, 39–41].

Identifying promising lines for various uses, from industry to medicine, is challenging and remarkable. Superior flax lines identified in this study can be used for enhanced studies to breed new varieties with a wide range of potential customers in the food market and industry (Table S2). Oil content and its fatty acid composition are the main components of flax genotypes, essentially determining its value and market end-use for nutritional or industrial purposes [9, 42, 43]. Hence, modifying fatty acid profiles to improve oil quality is an important and evolving issue in flax research [9, 44]. Families 23B, 84B, 36B, 24B, 71B, 30B, 15B, 88B, 62B, 31Y, 27Y, 83Y, 51Y, 95Y, 70Y, 35Y, 63Y, and 87Y were the prominent ones with superior oil content. Choosing genotypes with high oil content is pivotal since improves the potential for sustainable production and increases farmers and industry profitability [42]. Families 43B, 73B, 17B, 34B, 64B, 21B, 62B, 82B, 33Y, 85Y, 42Y, 29Y, 65Y, 35Y, 91Y, 63Y, 47Y, 75Y, 20Y, and 83Y with high linolenic acid can be recommended for developing new varieties for eco-friendly paints, linoleum flooring, inks, and varnishes as well as for create functional foods fortified with omega-3 [42, 45]. Families 78B, 90B, 102B, 30B, 45B, 48B, 92B, 66B, 103B, 38B, 94B, 55B, 98B, 61Y, 99Y, 89Y, 7Y, 9Y, 14Y, 51Y, and 31Y with the lowest linolenic acid are less subject to rapid oxidation and thus more preferred as a cooking or salad oil. Considering that the actual amount of ɷ3/ɷ6 ratio in the diet of most societies, especially in Western countries, is < 1:10 [5, 46], the families 17B, 43B, 28B, 15B, 19B, 55B, 4B, 73B, 32B, 6B, 13B, 46B, 42Y, 18Y, 29Y, 33Y, 16Y, 27Y, 54Y, 85Y, 44Y, and 5Y with the highest ratio of ɷ3/ɷ6 acid provides a wonderful opportunity for balancing the ɷ3/ɷ6 ratio in human nutrition. High thermal stability is another critical indicator for vegetable oil used for frying and cooking, which can be met by producing highly monounsaturated and saturated oils [47]. Therefore, it is advisable to use seed oil of families 30B, 55B, 9Y, 16Y, 92B, 29Y, 54Y, 45B, 14Y, 17B, 4B, 73B, 15B, 8B, 27Y, 3Y, 7Y, 42Y, 18Y, and 13B with the highest ratio of oleic/unsaturated fatty acids for frying and cooking. The seed oil of families 8B, 64B, 62B, 88B, 34B, 21B, 69B, 24B, 66B, 73B, 59B, 68Y, 75Y, 61Y, 37Y, 20Y, 14Y, 70Y, 47Y, 33Y, and 7Y were rich in unsaturated fatty acids with a higher ratio of unsaturated/saturated fatty acids. Therefore, these families may serve as potential sources for producing highly unsaturated vegetable oils to meet consumers’ need to avoid saturated fats for their health [48].

Families 73B, 28B, 26B, 41B, 23B, 76B, 4B, 71B, 40Y, 51Y, 95Y, 42Y, 54Y, 70Y, 27Y, 5Y, 63Y, 72Y, and 75Y are suitable candidates for breeding programs, aiming to develop new varieties with increased protein content in the human diet. Despite containing essential amino acids, lysine is generally considered the limiting amino acid of flax protein [49, 50]. In this respect, families 94B, 32B, 45B, 6B, 46B, 86B, 64B, 82B, 55B, 77Y, 58Y, 31Y, 5Y, 44Y, and 56Y with high lysine content were notable starting the development of flaxseed protein-based foods to satisfy the amino acid nutritional requirements for the adults and babies. Families 34B, 23B, 74B, 60B, 19B, 87Y, 63Y, 89Y, 5Y, 77Y, and 47Y recorded the highest fiber content. These families are ideal for lowering total cholesterol levels and promoting heart health [50–53]. High levels of antioxidants are known to be suitable for the thermal and oxidative stability of vegetable oils [54, 55]. Families 23B, 84B, 36B, 24B, 71B, 30B, 15B, 88B, 62B, 31Y, 27Y, 83Y, 51Y, 95Y, 70Y, 35Y, 63Y, and 78B had high oxidative stability owing to a higher level of tocopherol content; it is necessary to mention that these families were also rich in phenolic content, which reduces the oxidation rate of oil. Lignans, the natural antioxidants in flax oil, prevent oxidative rancidity, provide high stability, and improve the oil’s shelf life [56]. The health benefits of lignans are well established; it is crucial to select high lignan families 84B, 11B, 96B, 88B, 45B, 23B, 78B, 13B, 30B, 76B, 90B, 35Y, 7Y, 91Y, 68Y, 22Y, and 95Y for their suitability for edible consumption, therapeutic and industrial applications [57]. Families 59B, 6B, 62B, 50B, 88B, 10B, 38B, 7Y, 79Y, 40Y, 42Y, 3Y, 20Y, and 83Y showed the highest mucilage values. Developing these families with high mucilage content allows farmers to reach a broader food, feed, pharmaceutical, and beauty market [42, 58, 59].

From a genetic point of view, seed-related characteristics such as seed coat color are the primary quality determinant of flax. The results showed that the seed color variations contributed differentially to the changes in the contents of biochemical components (Table S3), indicating that seed coat color should be considered an essential factor in the nutritional components of flaxseed. Interestingly, transcriptomics analysis on flax seeds has identified that transcriptional regulations of oil, fatty acids, lignans, flavonoids, and mucilage metabolism could occur in the seed coat during seed development [60, 61]. Higher amounts of palmitic acid and saturated fatty acids in brown seeds confer the property of oil thermo-oxidative stability during frying. Significantly higher contents of protein, fiber, tocopherol, phenolics, SDG, and SECO lignans in brown-seeded families indicated good quality and health benefits of the brown seeds. High levels of SDG and SECO in brown families can be due to the intensification of lignans biosynthesis in their seed coat compared to tannins, anthocyanins, and proanthocyanins. The higher antioxidant activity of brown flaxseed than yellow flaxseed may be due to the higher content of phenolic compounds such as lignans. Since lignans are a type of polyphenol, a high amount of lignans in brown-seeded families may enhance their ability to withstand drought and increase seed yield in water stress conditions [57, 62]. A higher composition of linoleic acid, linolenic acid, and unsaturated/saturated fatty acids in the studied yellow-seeded families makes these families healthier than the brown-seeded types. These results were coherent with earlier studies [13, 20, 32, 63] that indicated yellow-seeded genotypes tended to have higher linolenic acid and unsaturated/saturated fatty acids. Considering mucilage, yellow-seeded families expressed higher emulsifying capacity than brown families, making them more appropriate for pharmaceutical, medicinal, and cosmetic applications. No appreciable differences were found between yellow and brown seeds regarding the oil and dispensable amino acids methionine, isoleucine, histidine, and arginine, indicating that it is possible to breed lines with any yellow or brown seed coat color from this F6 generation to have high or low values of these traits. Similarly, in Morris’s study [64], no significant difference was reported between arginine, histidine, isoleucine, methionine, and oil content of yellow versus brown-seeded genotypes.

Examination of the PCA loading plot over the two years of cultivation made it possible to differentiate all 100 F6 families, of which brown and yellow-seeded families occupied separate positions. Families 73B, 17B, 43B, 34B, 30B, 80B, 98B, 4B, 64B, 55B, 86B, 32B, 82B, 41B, 6B, 60B, 8B, 46B, 21B, 50B, 19B, 79B, 69B, and 107B (all brown-seeded) occupied region I of the biplot had high saturated fatty acids and oleic/unsaturated fatty acid. This points to the high thermal stability of the oil in these families. Brown-seeded families 103B, 90B, 11B, 74B, 45B, 92B, 84B, 23B, 76B, 36B, 28B, 94B, 15B, 78B, 38B, 102B, 96B, 100B, 52B, 13B, 1B, 71B, 62B, 66B, 10B, 88B, 2B, 59B, 24B, 26B, 57B, 105B, 48B, and 106B located in the region II of the biplot with high oil, protein, lignan, tocopherol, and phenolic compounds had high nutritional value. Therefore, using brown flax in the diet could increase the intake of health-promoting compounds. Families 42Y, 33Y, 29Y, 85Y, 35Y, 91Y, 22Y, 77Y, 5Y, 56Y, 18Y, 54Y, 65Y, 9Y, 79Y, 14Y, 40Y, 101Y, 37Y, 58Y, 75Y, 20Y, 47Y, 72Y, 49Y, and 68Y (all yellow-seeded) located in the region III of the biplot had high linolenic acid, unsaturated/saturated fatty acids, and mucilage. Yellow-seeded families 27Y, 95Y, 31Y, 51Y, 16Y, 89Y, 87Y, 63Y, 70Y, 14Y, 7Y, 3Y, 83Y, 61Y, 97Y, and 99Y located in the region IV of the biplot with high linoleic content. As a result, yellow-seeded families may be desirable to develop healthy functional flax varieties with increased omega-3, omega-6, and mucilage content.

In addition to the fact that genetic factors influence the chemical composition of flax seeds, it is also affected by environmental conditions. Variations in year lead to variations in climatic variables such as temperature, solar radiation, precipitation, elevation, and soil nutrients that affect plant growth and result in different seed compositions [65, 66]. According to the meteorological data at our experimental site, plants experienced dryer conditions in the second year due to higher temperatures and lower relative humidity, leading to higher pan evaporation in 2022 (Table 2). Although the cultivation year affected almost all traits, ɷ3/ɷ6, lignans, oleic acid, linolenic acid, and essential amino acids were the most critical characteristics affected by year. In contrast, the ratio of unsaturated to saturated fatty acids, palmitic acid, and stearic acid was stable (Table 3). The results of the present study demonstrated that higher temperatures and less relative humidity during the flax growth period led to higher content of protein, fiber, oleic acid, linoleic acid, oleic acid/unsaturated fatty acids, total phenolics, tocopherol, mucilage, and essential amino acids. In contrast, the content of oil, linolenic acid, omega3/6, and lignans decreased with increasing temperature and reducing relative humidity. A similar result was observed by Quin et al. [67], in which the contents of total protein and most of the amino acids showed an increasing trend with increasing temperature, while the contents of total oil and linolenic acid showed a reverse trend. In plants, lipid metabolism and remodeling are essential short-term adaptive reactions for survival under temperature stress [68–70].Disturbance in the mechanism of seed photosynthesis, imperfect incorporation of carbohydrates into triacylglycerol, and transcriptional deregulation of the fatty acid biosynthesis pathway are probably the main reasons for less oil accumulation due to heat stress [7]. It is also reported that supra-optimal temperatures negatively impact polyunsaturated fatty acids content in plants; consequently, during higher temperatures, the polyunsaturated fatty acid content of oils decreases in favor of saturated fatty acids and mono-unsaturated fatty acids and decreases in the ɷ3/6 ratio [71–73]. The two integral membrane-bound fatty acid desaturases (FAD2 and FAD3) produce polyunsaturated fatty acids in oil crops. FAD2 converts oleic acid to linoleic acid, and subsequently, FAD3 modifies linoleic acid to linolenic acid [74, 75]. Based on the literature review, temperature can disrupt desaturase enzyme activity, i.e., oleic and linoleic desaturases [76, 77]. Some studies showed a significant up-regulation of *FAD2* or *FAD3* isogenes at a lower temperature, whereas others observed either down-regulation or no appreciable change [75, 78, 79]. In response to stress, plants exhibit an increased synthesis of phenolic compounds that provide essential physiological and ecological duties, mainly protecting against different types of stress [80]. Amino acids are fundamental small molecules for synthesizing structural and stress proteins. They can be used as osmoprotectants to overcome abiotic stress [81]. High temperature affects the upregulated expression of corresponding enzyme genes, leading to synthesizing and transferring the corresponding amino acid components [82]. Our results indicate that flax exposure to high temperatures leads to an increase in the total amino acid content and the reduction of damage caused by high temperatures.

A positive correlation between protein and oil content (Table S4) suggested that the F6 flax families can modify the relationship between the traits to reduce the intensity of improvement of one trait while reducing the other and simultaneously increasing both protein and oil content. The results obtained in the present study confirm that high oil content leads to high fiber content, which tends to increase tocopherol, phenolics, and lignans. Considering the obtained results, the selection to increase oil content would affect flax quality. The metabolic pathways of fatty acids may be important genetic factors responsible for the correlations among the components of the fatty acid profile [84]. Regarding the correlations among the components of the fatty acid profile, the strong inverse but significant correlations of oleic acid with linoleic and linolenic fatty acids indicated an antagonistic relationship between these components of flaxseed oil. According to Gomes et al. [83], this inverse relationship may be related to the action of *FAD2* during the biosynthesis of these fatty acids. The negative relationships of linolenic acid with linoleic acid and oleic acid with linoleic and linolenic acid can be best understood by the fatty acid biosynthesis pathway. Both linoleic and linolenic acids share a common biosynthesis pathway, so one can be increased at the cost of the other [28, 84]. A potential explanation for the positive association between palmitic and stearic acids could be attributed to the *GmFATB1a* gene, which, in addition to encoding palmitate thioesterase, has activities toward stearoyl acyl carrier protein substrates [85]. Positive relationships among qualitative indices like unsaturated/saturated fatty acids and tocopherol and oil content suggested that a higher amount of oil might be associated with higher oil’s nutritional properties.

Several routes are involved in the biosynthesis of antioxidants in plants, some of which are common among different antioxidant biosynthetic pathways. The shikimate pathway is responsible for synthesizing the tocopherol aromatic ring, and is the same pathway used by plants to synthesize secondary metabolites like phenolic compounds [86, 87]. Thus, some of the genes involved in the shikimate pathway could have a possible pleiotropic effect on tocopherol and phenolics [88] and might be an illustrative reason for the robust genetic correlation between the two traits.

A positive correlation between linolenic and linoleic acid contents and total tocopherol content suggests a biochemical link between the tocopherol levels and the degree of unsaturation in flax oil. Given the high levels of polyunsaturated fatty acids in oilseeds, oxidative degradation has more potential. Our discovery of high tocopherol levels in oils rich in polyunsaturated fatty acids is promising, as tocopherol can act as radical scavengers and are involved in pre-oxidation reactions [89]. The significant positive correlation between protein content and essential amino acids is also promising, as high protein content families tend to have high contents of all essential amino acids simultaneously. Strong correlations between amino acids were expected since these traits may share some common genetic regulatory factors and enzymes belonging to the same biosynthetic pathways [90].

## Conclusion

Our study showed that flax nutritional components depended on genetic predisposition, followed by metrological indicators, especially mean daily temperatures and relative humidity. The biochemical composition varied greatly between F6 families, especially the profile of fatty acids and the accumulation of bioactive compounds. Based on the properties discussed, it is possible to determine the best grade of flax for the food, cosmetic, and pharmaceutical industries. Due to the vast application range of flax in industries, animal feeding, and medicine, families 84B, 23B, 95Y, 31Y, 51Y, 71B, 76B, 74B, 7Y, 48B, 89Y, 16Y, 103B, 3Y, 13B, 15B, 94B, 24B, and 45B were the most preferred options. Using genetically improved low- and high-linolenic acid flax families could be a more cost-effective and nutritionally beneficial approach for food and industrial applications. Chemical profiling and characterization of phenolic acids, tocopherols, and lignans obtained in the present study can provide a platform for selecting families 84B, 23B, 35Y, 95Y, 30B, 88B, and 78B as the sources of antioxidants for replacing synthetic compounds. The positive relationship among bioactive compounds provides a basal platform for breeding and agronomic programs to simultaneously focus on improving multiple nutritional and phytochemical characteristics. The contents of protein, fiber, oleic acid, linoleic acid, oleic acid/unsaturated fatty acids, total phenolics, tocopherol, mucilage, and essential amino acids showed an increasing trend with increasing average temperatures and decreasing relative humidity. In contrast, the content of oil, linolenic acid, omega-3/6, and lignans showed a reverse trend. In addition, our study highlighted that seed coat color is a significant player influencing the biochemical composition of flax. Taken together, our results implied that brown-seeded flax is a credible resource for producing more protein, fiber, and antioxidant compounds, particularly tocopherol, phenolics, and lignans, which can be used in food additives and pharmaceutics industries to strengthen nutrition and human health. Remarkably, a high level of polyunsaturated fatty acids and mucilage of yellow-seeded flax makes the oil nutritionally and therapeutically valuable for culinary purposes.

### Electronic supplementary material

Below is the link to the electronic supplementary material.

Supplementary Material 1

## Data Availability

The data presented in this study are available in the article and tables.
