# Supplementary material for: Quality assessment of flax advanced breeding lines varying in seed coat color and their potential use in the food and industrial applications
Source: BMC Plant Biol. 2024 Jan 23;24:60. doi: 10.1186/s12870-024-04733-1 (PMC10804595; doi:10.1186/s12870-024-04733-1)
Supplement: Supplementary file 1 — Supplementary Material 1 [file 12870_2024_4733_MOESM1_ESM.docx]

| **Table S1.** Information on F6 families used in this study. | | | | | | | | | | | |
| --- | --- | --- | --- | --- | --- | --- | --- | --- | --- | --- | --- |
| Family  number | cross | seed  color | |  | Family  number | cross | seed  color |  | Family  number | cross | seed  color |
| 1 | 3×1 | | b |  | 43 | 2×7 | b |  | 85 | 5×3 | y |
| 2 | 2×4 | | b |  | 44 | 8×7 | y |  | 86 | 5×3 | b |
| 3 | 8×4 | | y |  | 45 | 8×7 | b |  | 87 | 4×5 | y |
| 4 | 8×4 | | b |  | 46 | 4×1 | b |  | 88 | 4×5 | b |
| 5 | 5×6 | | y |  | 47 | 6×3 | y |  | 89 | 6×7 | y |
| 6 | 5×6 | | b |  | 48 | 6×3 | b |  | 90 | 6×7 | b |
| 7 | 4×7 | | y |  | 49 | 7×4 | y |  | 91 | 7×8 | y |
| 8 | 4×7 | | b |  | 50 | 7×4 | b |  | 92 | 7×8 | b |
| 9 | 7×1 | | y |  | 51 | 1×6 | y |  | 94 | 2×3 | b |
| 10 | 7×1 | | b |  | 52 | 1×6 | b |  | 95 | 8×3 | y |
| 11 | 1×3 | | b |  | 54 | 3×7 | y |  | 96 | 8×3 | b |
| 13 | 3×2 | | b |  | 55 | 3×7 | b |  | 97 | 5×4 | y |
| 14 | 2×5 | | y |  | 56 | 2×8 | y |  | 98 | 5×4 | b |
| 15 | 2×5 | | b |  | 57 | 2×8 | b |  | 99 | 4×6 | y |
| 16 | 8×5 | | y |  | 58 | 5×1 | y |  | 100 | 4×6 | b |
| 17 | 8×5 | | b |  | 59 | 5×1 | b |  | 101 | 6×8 | y |
| 18 | 5×7 | | y |  | 60 | 4×2 | b |  | 102 | 6×8 | b |
| 19 | 5×7 | | b |  | 61 | 6×4 | y |  | 103 | 1×2 | b |
| 20 | 4×8 | | y |  | 62 | 6×4 | b |  | 104 | 3×5 | y |
| 21 | 4×8 | | b |  | 63 | 7×5 | y |  | 105 | 3×5 | b |
| 22 | 7×2 | | y |  | 64 | 7×5 | b |  | 106 | 6×2 | y |
| 23 | 7×2 | | b |  | 65 | 1×7 | y |  | 107 | 6×2 | b |
| 24 | 1×4 | | b |  | 66 | 1×7 | b |  |  |  |  |
| 26 | 3×4 | | b |  | 68 | 3×8 | y |  |  |  |  |
| 27 | 2×6 | | y |  | 69 | 3×8 | b |  |  |  |  |
| 28 | 2×6 | | b |  | 70 | 8×1 | y |  |  |  |  |
| 29 | 8×6 | | y |  | 71 | 8×1 | b |  |  |  |  |
| 30 | 8×6 | | b |  | 72 | 5×2 | y |  |  |  |  |
| 31 | 5×8 | | y |  | 73 | 5×2 | b |  |  |  |  |
| 32 | 5×8 | | b |  | 74 | 4×3 | b |  |  |  |  |
| 33 | 6×1 | | y |  | 75 | 6×5 | y |  |  |  |  |
| 34 | 6×1 | | b |  | 76 | 6×5 | b |  |  |  |  |
| 35 | 7×3 | | y |  | 77 | 7×6 | y |  |  |  |  |
| 36 | 7×3 | | b |  | 78 | 7×6 | b |  |  |  |  |
| 37 | 1×5 | | y |  | 79 | 1×8 | y |  |  |  |  |
| 38 | 1×5 | | b |  | 80 | 1×8 | b |  |  |  |  |
| 40 | 3×6 | | y |  | 82 | 2×1 | b |  |  |  |  |
| 41 | 3×6 | | b |  | 83 | 8×2 | y |  |  |  |  |
| 42 | 2×7 | | y |  | 84 | 8×2 | b |  |  |  |  |

| **Table S2.** Average values of 19 traits evaluated in 108 flax genotypes (100 F6 families and 8 parental genotypes) during two years. |
| --- |

| Family | oil  (%) | PRO  (g/kg) | FIB  (%) | OLE  (%) | LIO  (%) | LIN  (%) | PAL  (%) | STR  (%) | ASP  (%) | MET  (%) | ISOLEU  (%) | LEU  (%) | LYS  (%) | HIS  (%) | ARG  (%) | omega 3/6 | USAT/SAT | OLE/USAT | TTC  (mg/100g OE) | TPC  (mg GAE/g OE) | SDG  (mg/g) | SECO  (mg/g) | | MUC  (%) | |
| --- | --- | --- | --- | --- | --- | --- | --- | --- | --- | --- | --- | --- | --- | --- | --- | --- | --- | --- | --- | --- | --- | --- | --- | --- | --- |
| 1 | 52.70 | 156.70 | 10.21 | 37.31 | 27.71 | 28.79 | 10.46 | 9.46 | 0.34 | 0.02 | 1.36 | 1.92 | 0.90 | 0.36 | 0.16 | 1.04 | 4.71 | 0.40 | 41.11 | 15.62 | 14.80 | | 7.80 | | 9.73 |
| 2 | 54.33 | 167.05 | 12.37 | 38.08 | 30.88 | 24.21 | 8.45 | 7.45 | 0.62 | 0.04 | 1.38 | 2.21 | 1.19 | 0.33 | 0.29 | 0.78 | 5.86 | 0.41 | 42.38 | 16.10 | 10.80 | | 3.80 | | 10.60 |
| 3 | 48.75 | 182.95 | 10.03 | 36.65 | 25.68 | 25.87 | 8.70 | 7.70 | 0.87 | 0.02 | 1.30 | 1.65 | 0.97 | 0.66 | 0.24 | 1.01 | 5.38 | 0.42 | 38.03 | 14.45 | 12.50 | | 5.50 | | 11.30 |
| 4 | 38.61 | 224.85 | 10.36 | 36.88 | 21.43 | 26.18 | 9.61 | 8.61 | 1.18 | 0.10 | 1.42 | 2.05 | 1.34 | 0.89 | 0.55 | 1.22 | 4.64 | 0.44 | 30.12 | 11.44 | 14.30 | | 7.30 | | 9.87 |
| 5 | 31.48 | 226.20 | 12.93 | 32.45 | 24.25 | 27.51 | 11.08 | 10.08 | 1.00 | 0.09 | 1.91 | 1.93 | 1.65 | 0.94 | 0.49 | 1.13 | 3.98 | 0.39 | 24.55 | 9.33 | 12.80 | | 5.80 | | 9.71 |
| 6 | 39.58 | 197.65 | 12.38 | 36.32 | 24.13 | 28.22 | 10.23 | 9.23 | 1.02 | 0.08 | 1.97 | 1.88 | 1.77 | 1.42 | 0.48 | 1.17 | 4.56 | 0.41 | 30.87 | 11.73 | 11.90 | | 4.90 | | 11.32 |
| 7 | 42.70 | 192.85 | 10.94 | 39.93 | 36.12 | 20.26 | 8.48 | 7.48 | 0.62 | 0.10 | 1.25 | 2.86 | 0.82 | 0.69 | 0.15 | 0.56 | 6.04 | 0.41 | 33.31 | 12.66 | 15.40 | | 8.40 | | 11.50 |
| 8 | 49.70 | 160.15 | 12.28 | 53.24 | 38.16 | 33.08 | 6.91 | 5.91 | 0.83 | 0.07 | 1.55 | 1.76 | 0.80 | 0.80 | 0.13 | 0.87 | 9.72 | 0.43 | 38.77 | 14.73 | 14.25 | | 7.25 | | 10.71 |
| 9 | 26.00 | 144.75 | 7.72 | 38.73 | 23.00 | 20.34 | 14.42 | 13.42 | 0.59 | 0.07 | 1.30 | 1.30 | 0.85 | 0.80 | 0.18 | 0.88 | 2.95 | 0.47 | 20.28 | 7.71 | 14.10 | | 7.10 | | 10.24 |
| 10 | 33.18 | 143.65 | 10.36 | 31.30 | 43.00 | 28.27 | 15.91 | 14.91 | 0.56 | 0.05 | 1.49 | 1.78 | 0.89 | 0.31 | 0.45 | 0.66 | 3.33 | 0.31 | 25.88 | 9.83 | 14.15 | | 7.15 | | 11.05 |
| 11 | 45.08 | 146.35 | 11.86 | 40.79 | 38.67 | 26.27 | 17.03 | 16.03 | 0.90 | 0.04 | 1.94 | 2.10 | 0.90 | 0.30 | 0.32 | 0.68 | 3.20 | 0.39 | 35.16 | 13.36 | 16.10 | | 9.10 | | 10.25 |
| 13 | 49.68 | 201.40 | 9.87 | 35.04 | 23.51 | 26.81 | 8.58 | 7.58 | 0.47 | 0.05 | 1.35 | 1.83 | 0.89 | 0.39 | 0.26 | 1.14 | 5.29 | 0.41 | 38.75 | 14.73 | 15.25 | | 8.25 | | 10.55 |
| 14 | 54.85 | 167.70 | 10.82 | 34.69 | 23.63 | 20.61 | 6.37 | 5.37 | 0.83 | 0.08 | 1.47 | 1.78 | 1.30 | 0.32 | 0.40 | 0.87 | 6.72 | 0.44 | 42.78 | 16.26 | 12.40 | | 5.40 | | 9.17 |
| 15 | 60.28 | 196.25 | 10.36 | 36.01 | 20.12 | 27.66 | 10.17 | 9.17 | 0.82 | 0.06 | 1.44 | 1.87 | 1.20 | 0.72 | 0.44 | 1.37 | 4.33 | 0.43 | 47.01 | 17.87 | 13.80 | | 6.80 | | 10.18 |
| 16 | 56.03 | 197.65 | 10.44 | 38.95 | 18.09 | 27.14 | 12.07 | 11.07 | 1.01 | 0.03 | 1.40 | 2.00 | 1.29 | 0.91 | 0.42 | 1.50 | 3.64 | 0.46 | 43.70 | 16.61 | 13.00 | | 6.00 | | 10.25 |
| 17 | 38.25 | 171.80 | 10.47 | 54.99 | 21.88 | 48.46 | 13.99 | 12.99 | 3.08 | 0.04 | 3.73 | 5.73 | 0.88 | 4.68 | 0.52 | 2.21 | 4.65 | 0.44 | 29.84 | 11.34 | 14.00 | | 7.00 | | 10.30 |
| 18 | 35.05 | 204.35 | 11.57 | 34.39 | 18.30 | 31.03 | 12.36 | 11.36 | 0.70 | 0.04 | 1.52 | 1.87 | 1.04 | 1.04 | 0.33 | 1.70 | 3.53 | 0.41 | 27.34 | 10.39 | 11.90 | | 4.90 | | 9.48 |
| 19 | 27.00 | 136.70 | 13.01 | 33.92 | 21.06 | 27.98 | 12.05 | 11.05 | 0.72 | 0.05 | 1.44 | 1.82 | 0.96 | 0.87 | 0.25 | 1.33 | 3.59 | 0.41 | 21.06 | 8.00 | 10.80 | | 3.80 | | 10.77 |
| 20 | 45.46 | 172.20 | 10.41 | 39.42 | 38.74 | 34.51 | 8.47 | 7.47 | 1.38 | 0.04 | 1.56 | 1.73 | 0.83 | 0.64 | 0.28 | 0.89 | 7.07 | 0.35 | 35.45 | 13.47 | 13.10 | | 6.10 | | 11.10 |
| 21 | 37.43 | 187.90 | 10.93 | 34.38 | 37.90 | 37.32 | 8.31 | 7.31 | 1.25 | 0.10 | 1.42 | 2.02 | 0.93 | 0.90 | 0.30 | 0.98 | 7.02 | 0.31 | 29.20 | 11.09 | 13.80 | | 6.80 | | 10.84 |
| 22 | 47.88 | 145.66 | 11.36 | 37.24 | 41.21 | 28.59 | 16.03 | 15.03 | 1.76 | 0.09 | 1.51 | 1.66 | 0.76 | 0.70 | 0.40 | 0.69 | 3.45 | 0.35 | 37.34 | 14.19 | 15.20 | | 8.20 | | 10.58 |
| 23 | 84.35 | 247.70 | 14.58 | 28.92 | 43.06 | 27.22 | 15.21 | 14.21 | 0.95 | 0.02 | 1.50 | 1.80 | 0.82 | 0.50 | 0.47 | 0.63 | 3.37 | 0.29 | 65.79 | 25.00 | 14.80 | | 7.80 | | 10.28 |
| 24 | 68.08 | 202.95 | 11.94 | 37.61 | 30.85 | 32.68 | 8.34 | 7.34 | 0.76 | 0.05 | 2.00 | 3.32 | 0.87 | 0.57 | 0.36 | 1.06 | 6.45 | 0.37 | 53.10 | 20.18 | 14.10 | | 7.10 | | 10.55 |
| 26 | 57.85 | 279.50 | 9.07 | 30.69 | 25.68 | 24.50 | 8.41 | 7.41 | 1.14 | 0.10 | 1.50 | 2.70 | 1.11 | 0.62 | 0.61 | 0.95 | 5.11 | 0.38 | 45.12 | 17.15 | 12.40 | | 5.40 | | 10.77 |
| 27 | 70.70 | 227.05 | 11.34 | 32.91 | 18.06 | 26.49 | 12.44 | 11.44 | 0.92 | 0.04 | 1.57 | 2.16 | 1.28 | 0.74 | 0.53 | 1.47 | 3.24 | 0.42 | 55.15 | 20.96 | **11.80** | | 4.80 | | 9.29 |
| 28 | 50.43 | 281.85 | 10.82 | 32.56 | 21.10 | 29.41 | 14.63 | 13.63 | 0.85 | 0.02 | 1.60 | 2.47 | 1.21 | 0.57 | 0.49 | 1.39 | 2.94 | 0.39 | 39.34 | 14.95 | 12.90 | | 5.90 | | 10.19 |
| 29 | 34.30 | 131.85 | 10.05 | 60.84 | 26.91 | 45.49 | 13.39 | 12.39 | 3.06 | 0.08 | 2.47 | 5.71 | 1.02 | 4.22 | 0.49 | 1.69 | 5.17 | 0.46 | 26.75 | 10.17 | 14.30 | | 7.30 | | 10.89 |
| 30 | 60.43 | 139.40 | 10.08 | 48.89 | 22.80 | 15.66 | 14.29 | 13.29 | 1.64 | 0.08 | 2.72 | 4.68 | 0.89 | 0.82 | 0.50 | 0.69 | 3.17 | 0.56 | 47.13 | 17.91 | 15.00 | | 8.00 | | 10.00 |
| 31 | 71.28 | 207.70 | 11.63 | 30.29 | 23.05 | 23.10 | 11.87 | 10.87 | 1.19 | 0.07 | 1.48 | 2.11 | 1.67 | 0.80 | 0.51 | 1.00 | 3.36 | 0.40 | 55.59 | 21.13 | 13.70 | | 6.70 | | 10.83 |
| 32 | 48.73 | 128.30 | 11.20 | 31.29 | 23.52 | 28.05 | 11.74 | 10.74 | 1.25 | 0.07 | 1.49 | 2.32 | 1.80 | 0.69 | 0.52 | 1.19 | 3.69 | 0.38 | 38.01 | 14.44 | **11.10** | | 4.10 | | 10.42 |
| 33 | 33.08 | 124.50 | 11.51 | 49.69 | 43.30 | 67.46 | 13.63 | 12.63 | 2.92 | 0.18 | 3.42 | 7.23 | 0.70 | 4.81 | 0.41 | 1.56 | 6.11 | 0.31 | 25.80 | 9.80 | 13.85 | | 6.85 | | 9.17 |
| 34 | 38.00 | 129.00 | 14.80 | 59.64 | 49.05 | 43.10 | 10.86 | 9.86 | 3.03 | 0.09 | 1.99 | 7.74 | 0.46 | 4.34 | 0.46 | 0.88 | 7.33 | 0.39 | 29.64 | 11.26 | 12.30 | | 5.30 | | 9.62 |
| 35 | 63.45 | 139.20 | 9.19 | 52.11 | 43.54 | 43.15 | 12.71 | 11.71 | 1.31 | 0.15 | 2.51 | 4.88 | 0.49 | 4.54 | 0.48 | 0.99 | 5.68 | 0.38 | 49.49 | 18.81 | 15.50 | | 8.50 | | 9.99 |
| 36 | 68.83 | 152.85 | 9.20 | 41.54 | 45.07 | 25.32 | 13.84 | 12.84 | 2.29 | 0.08 | 1.34 | 1.55 | 0.85 | 0.69 | 0.21 | 0.56 | 4.20 | 0.37 | 53.68 | 20.40 | 12.40 | | 5.40 | | 10.17 |
| 37 | 52.35 | 157.53 | 10.97 | 34.39 | 34.56 | 23.79 | 6.69 | 5.69 | 0.81 | 0.08 | 3.06 | 1.91 | 0.81 | 0.69 | 0.31 | 0.69 | 7.49 | 0.37 | 40.83 | 15.52 | 13.10 | | 6.10 | | 10.77 |
| 38 | 40.50 | 187.20 | 9.63 | 34.88 | 35.94 | 21.96 | 19.86 | 18.86 | 0.58 | 0.07 | 2.64 | 2.28 | 0.75 | 0.54 | 0.38 | 0.61 | 2.40 | 0.38 | 31.59 | 12.00 | 11.80 | | 4.80 | | 11.05 |
| 40 | 45.75 | 262.90 | 8.87 | 31.71 | 26.18 | 27.66 | 12.66 | 11.66 | 1.55 | 0.09 | 1.52 | 2.76 | 1.18 | 0.57 | 0.58 | 1.06 | 3.52 | 0.37 | 35.69 | 13.56 | 10.90 | | 3.90 | | 11.37 |
| 41 | 40.04 | 247.75 | 8.80 | 33.95 | 28.08 | 28.10 | 9.30 | 8.30 | 1.64 | 0.07 | 1.52 | 2.50 | 1.18 | 0.55 | 0.61 | 1.00 | 5.12 | 0.38 | 31.23 | 11.87 | 13.30 | | 6.30 | | 10.00 |
| 42 | 40.57 | 229.85 | 9.98 | 54.44 | 21.50 | 55.60 | 13.14 | 12.14 | 2.40 | 0.16 | 3.77 | 7.12 | 1.13 | 0.60 | 0.21 | 2.59 | 5.20 | 0.41 | 31.64 | 12.02 | 11.80 | | 4.80 | | 11.36 |
| 43 | 27.94 | 159.85 | 11.88 | 49.34 | 31.14 | 59.74 | 17.84 | 16.84 | 2.50 | 0.22 | 2.52 | 7.16 | 0.72 | 0.66 | 0.22 | 1.92 | 4.04 | 0.35 | 21.79 | 8.28 | 11.20 | | 4.20 | | 10.94 |
| Family | oil  (%) | PRO  (g/kg) | FIB  (%) | OLE  (%) | LIO  (%) | LIN  (%) | PAL  (%) | STR  (%) | ASP  (%) | MET  (%) | ISOLEU  (%) | LEU  (%) | LYS  (%) | HIS  (%) | ARG  (%) | omega 3/6 | USAT/SAT | OLE/USAT | TTC  (mg/100g OE) | TPC  (mg GAE/g OE) | SDG  (mg/g) | | SECO  (mg/g) | | MUC  (%) |
| 44 | 40.44 | 193.05 | 11.05 | 32.12 | 23.39 | 28.39 | 9.50 | 8.50 | 1.16 | 0.08 | 1.89 | 2.05 | 1.59 | 0.62 | 0.21 | 1.21 | 4.66 | 0.38 | 31.54 | 11.99 | 13.30 | | 6.30 | | 10.86 |
| 45 | 48.08 | 207.70 | 11.45 | 32.81 | 24.28 | 15.74 | 9.05 | 8.05 | 0.85 | 0.07 | 2.22 | 1.92 | 1.77 | 0.78 | 0.27 | 0.65 | 4.26 | 0.45 | 37.50 | 14.25 | 15.40 | | 8.40 | | 9.99 |
| 46 | 33.85 | 140.76 | 11.08 | 30.51 | 23.82 | 26.70 | 10.11 | 9.11 | 0.84 | 0.05 | 1.38 | 2.61 | 1.70 | 0.56 | 0.29 | 1.12 | 4.22 | 0.38 | 26.40 | 10.03 | 11.90 | | 4.90 | | 10.47 |
| 47 | 39.38 | 152.25 | 12.40 | 33.09 | 46.03 | 35.29 | 9.72 | 8.72 | 0.76 | 0.03 | 1.36 | 2.59 | 0.63 | 0.57 | 0.33 | 0.77 | 6.20 | 0.29 | 30.72 | 11.67 | 13.70 | | 6.70 | | 9.50 |
| 48 | 40.88 | 145.20 | 9.47 | 28.95 | 41.03 | 16.39 | 8.85 | 7.85 | 0.57 | 0.04 | 1.34 | 2.28 | 0.56 | 0.74 | 0.30 | 0.40 | 5.17 | 0.34 | 31.89 | 12.12 | 14.80 | | 7.80 | | 10.95 |
| 49 | 36.05 | 137.40 | 8.61 | 36.94 | 39.69 | 32.85 | 15.33 | 14.33 | 1.58 | 0.06 | 1.59 | 1.76 | 0.72 | 0.97 | 0.28 | 0.83 | 3.69 | 0.34 | 28.12 | 10.68 | 11.55 | | 4.55 | | 10.68 |
| 50 | 31.56 | 128.55 | 9.28 | 29.94 | 39.54 | 29.15 | 16.60 | 15.60 | 3.08 | 0.06 | 1.44 | 1.60 | 0.90 | 0.93 | 0.26 | 0.74 | 3.06 | 0.30 | 24.62 | 9.35 | 11.10 | | 4.10 | | 11.14 |
| 51 | 67.95 | 238.03 | 9.20 | 26.45 | 42.06 | 21.40 | 24.57 | 23.57 | 0.60 | 0.10 | 1.87 | 1.84 | 0.81 | 0.48 | 0.42 | 0.51 | 1.87 | 0.29 | 53.00 | 20.14 | 13.10 | | 6.10 | | 10.77 |
| 52 | 43.23 | 151.35 | 8.20 | 22.87 | 37.11 | 26.85 | 20.27 | 19.27 | 0.99 | 0.05 | 1.71 | 2.04 | 1.03 | 0.25 | 0.44 | 0.72 | 2.20 | 0.26 | 33.72 | 12.81 | 14.70 | | 7.70 | | 10.56 |
| 54 | 30.38 | 228.30 | 9.89 | 39.00 | 20.55 | 26.59 | 9.59 | 8.59 | 0.75 | 0.09 | 1.31 | 2.03 | 0.96 | 0.62 | 0.26 | 1.29 | 4.74 | 0.45 | 23.70 | 9.00 | 13.40 | | 6.40 | | 10.66 |
| 55 | 34.53 | 125.65 | 9.37 | 38.06 | 17.54 | 22.62 | 9.21 | 8.21 | 0.99 | 0.09 | 1.27 | 1.75 | 1.38 | 0.90 | 0.29 | 1.29 | 4.49 | 0.49 | 26.93 | 10.23 | 14.60 | | 7.60 | | 9.49 |
| 56 | 40.68 | 137.60 | 11.64 | 32.20 | 26.05 | 27.28 | 10.47 | 9.47 | 0.98 | 0.11 | 1.36 | 1.90 | 1.39 | 0.86 | 0.37 | 1.05 | 4.29 | 0.38 | 31.73 | 12.06 | 13.90 | | 6.90 | | 10.40 |
| 57 | 55.80 | 142.85 | 9.26 | 33.18 | 25.83 | 26.98 | 11.83 | 10.83 | 1.10 | 0.10 | 1.36 | 1.98 | 1.24 | 0.74 | 0.33 | 1.04 | 3.79 | 0.39 | 43.52 | 16.54 | 13.10 | | 6.10 | | 10.94 |
| 58 | 39.88 | 179.15 | 12.05 | 28.82 | 38.92 | 29.89 | 9.80 | 8.80 | 1.25 | 0.06 | 2.02 | 1.57 | 1.76 | 0.44 | 0.42 | 0.77 | 5.25 | 0.30 | 31.10 | 11.82 | 13.80 | | 6.80 | | 10.26 |
| 59 | 48.90 | 180.25 | 10.39 | 41.93 | 39.72 | 29.54 | 9.48 | 8.48 | 1.05 | 0.06 | 2.04 | 2.40 | 0.73 | 0.42 | 0.38 | 0.74 | 6.19 | 0.38 | 38.14 | 14.49 | 13.50 | | 6.50 | | 11.36 |
| 60 | 29.88 | 138.80 | 13.07 | 33.05 | 36.90 | 29.04 | 12.23 | 11.23 | 1.76 | 0.06 | 1.51 | 3.14 | 0.79 | 0.77 | 0.37 | 0.79 | 4.22 | 0.33 | 23.30 | 8.85 | 14.50 | | 7.50 | | 10.18 |
| 61 | 51.44 | 153.35 | 9.42 | 29.20 | 58.96 | 16.03 | 6.78 | 5.78 | 0.51 | 0.08 | 1.39 | 4.83 | 0.59 | 0.48 | 0.24 | 0.27 | 8.30 | 0.28 | 40.12 | 15.25 | 13.90 | | 6.90 | | 10.03 |
| 62 | 58.73 | 156.80 | 8.46 | 47.69 | 60.15 | 35.44 | 9.00 | 8.00 | 0.66 | 0.02 | 1.77 | 1.36 | 0.54 | 0.50 | 0.18 | 0.59 | 8.43 | 0.33 | 45.81 | 17.41 | 14.70 | | 7.70 | | 11.31 |
| 63 | 63.01 | 222.00 | 13.52 | 32.03 | 55.98 | 37.34 | 12.52 | 11.52 | 3.10 | 0.06 | 1.49 | 1.35 | 0.76 | 0.69 | 0.28 | 0.67 | 5.21 | 0.26 | 49.15 | 18.68 | 13.35 | | 6.35 | | 10.65 |
| 64 | 55.35 | 193.90 | 11.47 | 45.65 | 50.10 | 41.48 | 8.58 | 7.58 | 2.85 | 0.08 | 1.47 | 1.55 | 1.63 | 0.89 | 0.26 | 0.83 | 8.50 | 0.33 | 43.17 | 16.41 | 14.20 | | 7.20 | | 10.49 |
| 65 | 41.65 | 141.70 | 10.33 | 38.38 | 54.55 | 44.24 | 12.95 | 11.95 | 1.22 | 0.12 | 1.35 | 1.55 | 0.88 | 0.67 | 0.34 | 0.81 | 5.51 | 0.28 | 32.49 | 12.35 | 14.20 | | 7.20 | | 9.87 |
| 66 | 41.60 | 140.20 | 9.15 | 39.68 | 58.95 | 19.54 | 9.67 | 8.67 | 1.25 | 0.05 | 1.46 | 2.17 | 0.99 | 0.40 | 0.35 | 0.33 | 6.45 | 0.34 | 32.44 | 12.33 | 14.00 | | 7.00 | | 10.86 |
| 68 | 32.07 | 160.70 | 9.53 | 40.70 | 34.51 | 31.85 | 6.88 | 5.88 | 0.74 | 0.08 | 1.55 | 1.39 | 0.88 | 0.48 | 0.22 | 0.92 | 8.40 | 0.38 | 25.01 | 9.51 | 15.30 | | 8.30 | | 10.62 |
| 69 | 35.57 | 157.10 | 10.04 | 29.32 | 41.19 | 33.73 | 7.97 | 6.97 | 0.50 | 0.05 | 1.50 | 1.74 | 1.27 | 0.34 | 0.30 | 0.82 | 6.98 | 0.28 | 27.74 | 10.54 | 9.40 | | 2.40 | | 10.03 |
| 70 | 65.57 | 227.40 | 9.53 | 30.35 | 44.25 | 30.49 | 8.84 | 7.84 | 0.74 | 0.08 | 1.51 | 1.90 | 0.94 | 0.43 | 0.30 | 0.69 | 6.30 | 0.29 | 51.14 | 19.43 | 13.40 | | 6.40 | | 10.07 |
| 71 | 61.73 | 218.90 | 9.54 | 32.02 | 47.52 | 24.50 | 10.58 | 9.58 | 0.50 | 0.05 | 1.59 | 1.90 | 0.80 | 0.48 | 0.31 | 0.52 | 5.16 | 0.31 | 48.15 | 18.30 | 14.30 | | 7.30 | | 10.62 |
| 72 | 37.07 | 218.05 | 11.88 | 37.88 | 43.05 | 33.91 | 10.20 | 9.20 | 0.74 | 0.04 | 2.15 | 2.03 | 0.78 | 0.68 | 0.51 | 0.79 | 5.92 | 0.33 | 28.91 | 10.99 | 11.20 | | 4.20 | | 10.03 |
| 73 | 55.23 | 295.85 | 9.93 | 68.83 | 40.13 | 48.90 | 13.01 | 12.01 | 3.11 | 0.05 | 3.76 | 5.85 | 1.25 | 0.85 | 0.30 | 1.22 | 6.31 | 0.44 | 43.08 | 16.37 | 12.70 | | 5.70 | | 10.76 |
| 74 | 55.05 | 207.00 | 13.42 | 44.37 | 41.97 | 31.54 | 12.54 | 11.54 | 1.42 | 0.06 | 1.85 | 2.05 | 1.10 | 0.76 | 0.45 | 0.75 | 4.90 | 0.38 | 42.94 | 16.32 | 14.50 | | 7.50 | | 10.84 |
| 75 | 50.27 | 215.20 | 8.92 | 49.89 | 61.67 | 34.54 | 9.29 | 8.29 | 0.74 | 0.04 | 2.24 | 1.61 | 0.55 | 0.71 | 0.42 | 0.56 | 8.32 | 0.34 | 39.21 | 14.90 | 13.50 | | 6.50 | | 10.21 |
| 76 | 49.93 | 245.35 | 11.43 | 46.04 | 48.10 | 25.20 | 10.98 | 9.98 | 0.61 | 0.03 | 1.50 | 2.07 | 0.89 | 0.62 | 0.44 | 0.52 | 5.70 | 0.39 | 38.94 | 14.80 | 15.00 | | 8.00 | | 9.61 |
| 77 | 32.90 | 120.25 | 12.49 | 39.55 | 54.91 | 23.60 | 14.99 | 13.99 | 1.90 | 0.07 | 2.62 | 2.45 | 1.82 | 0.78 | 0.24 | 0.43 | 4.07 | 0.33 | 25.66 | 9.75 | 13.70 | | 6.70 | | 9.91 |
| 78 | 34.83 | 119.35 | 8.95 | 33.09 | 40.53 | 11.50 | 22.50 | 21.50 | 2.03 | 0.07 | 1.44 | 2.81 | 0.93 | 0.69 | 0.23 | 0.28 | 1.93 | 0.39 | 27.17 | 10.32 | 15.30 | | 8.30 | | 10.78 |
| 79 | 36.85 | 142.35 | 8.42 | 38.58 | 54.08 | 27.95 | 22.58 | 21.58 | 0.95 | 0.07 | 2.23 | 2.28 | 0.94 | 1.06 | 0.47 | 0.52 | 2.73 | 0.32 | 28.74 | 10.92 | 12.80 | | 5.80 | | 11.40 |
| 80 | 42.55 | 142.45 | 11.00 | 45.42 | 53.35 | 25.23 | 19.80 | 18.80 | 1.90 | 0.10 | 1.49 | 2.01 | 1.31 | 0.93 | 0.32 | 0.47 | 3.21 | 0.37 | 33.19 | 12.61 | 14.70 | | 7.70 | | 10.50 |
| 82 | 36.70 | 150.35 | 9.92 | 34.58 | 43.15 | 35.35 | 18.20 | 17.20 | 0.99 | 0.05 | 1.44 | 3.62 | 1.59 | 0.31 | 0.43 | 0.82 | 3.20 | 0.31 | 28.63 | 10.88 | 13.58 | | 6.58 | | 10.52 |
| 83 | 70.00 | 181.75 | 9.45 | 32.55 | 47.86 | 34.40 | 10.19 | 9.19 | 1.31 | 0.04 | 1.36 | 2.43 | 0.88 | 0.46 | 0.42 | 0.72 | 5.92 | 0.28 | 54.60 | 20.75 | 10.50 | | 3.50 | | 11.09 |
| 84 | 84.35 | 176.60 | 10.12 | 39.84 | 44.64 | 28.45 | 15.43 | 14.43 | 2.41 | 0.06 | 1.52 | 3.08 | 0.85 | 0.42 | 0.35 | 0.64 | 3.78 | 0.35 | 65.79 | 25.00 | 16.70 | | 9.70 | | 10.80 |
| 85 | 35.55 | 164.35 | 8.08 | 51.83 | 47.62 | 58.83 | 25.68 | 24.68 | 3.40 | 0.06 | 2.79 | 7.35 | 0.47 | 0.57 | 0.27 | 1.24 | 3.14 | 0.33 | 27.73 | 10.54 | 14.00 | | 7.00 | | 10.86 |
| 86 | 30.79 | 167.75 | 8.98 | 29.29 | 42.75 | 27.10 | 13.80 | 12.80 | 3.19 | 0.06 | 2.02 | 2.64 | 1.68 | 0.58 | 0.21 | 0.63 | 3.73 | 0.30 | 24.02 | 9.13 | 15.90 | | 8.90 | | 10.89 |
| 87 | 58.75 | 153.15 | 15.22 | 33.70 | 40.66 | 30.33 | 13.24 | 12.24 | 2.07 | 0.04 | 1.41 | 3.13 | 1.04 | 0.62 | 0.47 | 0.75 | 4.11 | 0.32 | 45.83 | 17.41 | 12.60 | | 5.60 | | 10.88 |
| 88 | 59.83 | 139.48 | 10.01 | 42.58 | 57.51 | 32.05 | 8.39 | 7.39 | 2.68 | 0.05 | 1.48 | 1.92 | 0.79 | 0.52 | 0.30 | 0.56 | 8.37 | 0.32 | 46.66 | 17.73 | 13.30 | | 6.30 | | 11.06 |
| 89 | 49.66 | 187.90 | 13.51 | 52.10 | 59.20 | 18.05 | 11.23 | 10.23 | 1.32 | 0.06 | 1.90 | 2.65 | 1.03 | 0.65 | 0.26 | 0.30 | 6.03 | 0.40 | 38.73 | 14.72 | 13.50 | | 6.50 | | 10.25 |
| Family | oil  (%) | PRO  (g/kg) | FIB  (%) | OLE  (%) | LIO  (%) | LIN  (%) | PAL  (%) | STR  (%) | ASP  (%) | MET  (%) | ISOLEU  (%) | LEU  (%) | LYS  (%) | HIS  (%) | ARG  (%) | omega 3/6 | USAT/SAT | OLE/USAT | TTC  (mg/100g OE) | TPC  (mg GAE/g OE) | SDG  (mg/g) | | SECO  (mg/g) | | MUC  (%) |
| 90 | 46.20 | 185.30 | 10.23 | 42.79 | 56.15 | 13.13 | 14.84 | 13.84 | 2.20 | 0.07 | 1.89 | 2.20 | 0.75 | 0.99 | 0.28 | 0.23 | 3.91 | 0.38 | 36.04 | 13.69 | 14.90 | | 7.90 | | 9.87 |
| 91 | 36.03 | 152.70 | 11.95 | 43.03 | 48.08 | 40.02 | 18.35 | 17.35 | 3.60 | 0.10 | 1.62 | 1.47 | 0.66 | 0.55 | 0.15 | 0.83 | 3.67 | 0.33 | 28.10 | 10.68 | 15.35 | | 8.35 | | 9.80 |
| 92 | 57.23 | 178.80 | 10.01 | 55.23 | 47.97 | 16.50 | 18.68 | 17.68 | 1.14 | 0.03 | 1.69 | 1.97 | 0.66 | 0.49 | 0.24 | 0.34 | 3.29 | 0.46 | 44.64 | 16.96 | 12.30 | | 5.30 | | 10.11 |
| 94 | 46.50 | 171.15 | 11.41 | 39.11 | 38.36 | 22.39 | 16.23 | 15.23 | 1.01 | 0.05 | 1.81 | 2.67 | 1.88 | 0.46 | 0.35 | 0.58 | 3.17 | 0.39 | 36.27 | 13.78 | 14.00 | | 7.00 | | 10.49 |
| 95 | 67.05 | 231.90 | 10.41 | 34.28 | 43.64 | 28.17 | 15.49 | 14.49 | 1.07 | 0.07 | 2.37 | 2.62 | 0.90 | 0.65 | 0.43 | 0.65 | 3.54 | 0.32 | 52.30 | 19.87 | 15.10 | | 8.10 | | 10.69 |
| 96 | 41.25 | 119.15 | 11.36 | 34.99 | 30.26 | 26.58 | 10.39 | 9.39 | 1.07 | 0.06 | 1.28 | 2.69 | 0.91 | 0.67 | 0.35 | 0.88 | 4.64 | 0.38 | 32.18 | 12.23 | 16.00 | | 9.00 | | 10.61 |
| 97 | 44.40 | 192.75 | 10.95 | 27.54 | 35.68 | 24.88 | 10.14 | 9.14 | 1.32 | 0.05 | 2.37 | 2.37 | 1.14 | 0.60 | 0.33 | 0.70 | 4.57 | 0.31 | 34.63 | 13.16 | 13.50 | | 6.50 | | 9.96 |
| 98 | 33.48 | 136.35 | 9.95 | 32.74 | 37.33 | 23.26 | 25.68 | 24.68 | 1.01 | 0.05 | 2.68 | 1.67 | 0.66 | 0.51 | 0.30 | 0.62 | 1.85 | 0.35 | 26.11 | 9.92 | 12.20 | | 5.20 | | 10.33 |
| 99 | 50.75 | 153.00 | 8.01 | 40.02 | 52.31 | 17.56 | 18.15 | 17.15 | 1.35 | 0.11 | 1.30 | 2.60 | 0.64 | 0.44 | 0.26 | 0.34 | 3.11 | 0.36 | 39.59 | 15.04 | 13.40 | | 6.40 | | 10.54 |
| 100 | 38.13 | 170.00 | 9.45 | 34.09 | 53.53 | 27.37 | 18.42 | 17.42 | 1.69 | 0.04 | 1.28 | 2.51 | 0.65 | 0.41 | 0.22 | 0.51 | 3.21 | 0.30 | 29.74 | 11.30 | 14.20 | | 7.20 | | 10.63 |
| 101 | 37.94 | 192.35 | 9.90 | 31.99 | 35.97 | 25.57 | 15.19 | 14.19 | 1.00 | 0.10 | 1.50 | 1.13 | 0.60 | 0.50 | 0.49 | 0.71 | 3.18 | 0.34 | 29.59 | 11.25 | 13.80 | | 6.80 | | 9.56 |
| 102 | 37.38 | 136.90 | 11.07 | 30.20 | 50.86 | 13.50 | 17.54 | 16.54 | 1.06 | 0.05 | 2.85 | 2.30 | 0.57 | 0.76 | 0.33 | 0.27 | 2.77 | 0.32 | 29.16 | 11.08 | 11.90 | | 4.90 | | 10.18 |
| 103 | 45.60 | 151.35 | 11.14 | 41.90 | 47.00 | 21.63 | 22.64 | 21.64 | 1.39 | 0.07 | 2.39 | 2.14 | 0.86 | 0.76 | 0.26 | 0.46 | 2.50 | 0.38 | 35.57 | 13.52 | 14.65 | | 7.65 | | 10.57 |
| P2 | 33.04 | 145.15 | 9.47 | 35.32 | 36.50 | 23.55 | 12.11 | 11.11 | 0.91 | 0.10 | 1.44 | 2.35 | 1.08 | 0.51 | 0.21 | 0.65 | 4.11 | 0.37 | 25.77 | 9.79 | 12.50 | | 5.50 | | 10.00 |
| P1 | 58.35 | 159.90 | 13.46 | 34.04 | 37.82 | 28.33 | 19.29 | 18.29 | 0.84 | 0.03 | 1.79 | 2.12 | 1.05 | 0.45 | 0.23 | 0.75 | 2.67 | 0.34 | 45.51 | 17.29 | 14.90 | | 7.90 | | 10.83 |
| P8 | 79.45 | 183.20 | 13.87 | 43.54 | 57.08 | 34.20 | 16.94 | 15.94 | 0.94 | 0.05 | 2.82 | 2.83 | 0.73 | 0.72 | 0.45 | 0.60 | 4.10 | 0.32 | 61.97 | 23.55 | 18.70 | | 11.70 | | 10.73 |
| P7 | 43.38 | 142.95 | 8.72 | 39.53 | 67.02 | 3.69 | 20.79 | 19.79 | 1.24 | 0.04 | 1.34 | 2.31 | 0.37 | 0.33 | 0.46 | 0.06 | 2.72 | 0.36 | 33.83 | 12.86 | 23.70 | | 16.70 | | 10.75 |
| P6 | 40.61 | 134.25 | 12.58 | 37.34 | 43.92 | 29.14 | 10.57 | 9.57 | 0.90 | 0.07 | 1.32 | 2.12 | 0.59 | 0.42 | 0.29 | 0.66 | 5.48 | 0.34 | 31.68 | 12.04 | 25.50 | | 18.50 | | 9.65 |
| P5 | 37.15 | 191.48 | 11.04 | 29.38 | 24.97 | 27.75 | 11.27 | 10.27 | 0.87 | 0.07 | 1.51 | 2.31 | 0.92 | 0.52 | 0.55 | 1.11 | 3.81 | 0.36 | 28.97 | 11.01 | 17.60 | | 10.60 | | 10.46 |
| P4 | 51.41 | 262.05 | 12.83 | 30.37 | 25.31 | 27.84 | 10.68 | 9.68 | 0.99 | 0.08 | 1.83 | 2.25 | 1.73 | 0.56 | 0.56 | 1.10 | 4.10 | 0.36 | 40.10 | 15.24 | 11.75 | | 4.75 | | 10.12 |
| P3 | 36.38 | 208.05 | 10.72 | 31.32 | 26.48 | 26.54 | 9.02 | 8.02 | 0.88 | 0.04 | 1.50 | 2.08 | 1.47 | 0.36 | 0.47 | 1.00 | 4.95 | 0.37 | 28.37 | 10.78 | 18.70 | | 11.70 | | 10.35 |

OIL, oil content; PRO, protein content; FIB, fiber content; OLE, oleic; LIO, linoleic; LIN, linolenic; PAL, palmitic; STR, stearic; Omega 3/6 linolenic to linoleic ratio; USAT/SAT, unsaturated to saturated ratio; OLE/USAT, oleic to unsaturated ratio; TTC, total tocopherol content; TPC, total phenolic content; ASP, aspartic; MET, methionine; ISOLEU, isoleucine; LEU, leucine; LYS, lysine; HIST, histidine, SDG, secoisolariciresinol diglucoside; SECO, secoisolariciresinol; MUC, mucilage.

| **Table S3.** Comparison of mean values for traits evaluated in 100 F6 families of flax with yellow and brown seed coat colors. | | | | | | | | | |
| --- | --- | --- | --- | --- | --- | --- | --- | --- | --- |
|  | | |  | Seed color | | |  | | LSD (5%) |
| Trait | | |  | Brown |  | Yellow |  | |  |
| Oil (%) |  | 46.63±1.83 | | |  | 46.86±3.51 | |  | 5.47 |
| PRO (g/kg) |  | 197.88±20.29 | | |  | 172.20±14.57 | |  | 21.07 |
| FIB (%) |  | 13.97±0.32 | | |  | 9.20±0.36 | |  | 2.35 |
| OLE (%) |  | 28.09±2.15 | | |  | 22.84±2.75 | |  | 2.08 |
| LIO (%) |  | 35.03±2.66 | | |  | 39.88±2.41 | |  | 3.44 |
| LIN (%) |  | 30.80±1.60 | | |  | 42.49±1.52 | |  | 4.08 |
| PAL (%) |  | 18.09±1.75 | | |  | 11.73±1.37 | |  | 1.21 |
| STR (%) |  | 13.09±1.01 | | |  | 7.73±1.37 | |  | 1.02 |
| ASP (%) |  | 1.74±0.22 | | |  | 1.22±0.91 | |  | 0.47 |
| MET (%) |  | 0.10±0.42 | | |  | 0.06±0.19 | |  | 0.11 |
| ISOLE (%) |  | 1.94±0.02 | | |  | 1.50±0.02 | |  | 0.06 |
| LEU (%) |  | 2.83±0.26 | | |  | 2.18±0.31 | |  | 0.63 |
| LYS (%) |  | 1.15±0.48 | | |  | 0.94±0.57 | |  | 0.05 |
| HIS (%) |  | 0.93±0.07 | | |  | 0.84±0.06 | |  | 0.05 |
| ARG (%) |  | 0.44±0.05 | | |  | 0.35±0.02 | |  | 0.68 |
| Omega 3/6 |  | 1.24±0.01 | | |  | 1.81±0.01 | |  | 0.09 |
| USAT/SAT |  | 5.11±2.72 | | |  | 6.87±2.52 | |  | 1.05 |
| OLE/USAT |  | 0.47±0.72 | | |  | 0.37±0.37 | |  | 0.34 |
| TTC (mg/100g OE) |  | 37.37±1.72 | | |  | 30.55±1.91 | |  | 5.91 |
| TPC (mg GAE/g OE) |  | 15.82±0.01 | | |  | 9.89±0.01 | |  | 7.11 |
| SDG (mg/g) |  | 17.90±2.72 | | |  | 13.05±2.52 | |  | 3.86 |
| SECO (mg/g) |  | 8.99±0.72 | | |  | 6.05±0.37 | |  | 2.31 |
| MUC (%) |  | 9.48±1.72 | | |  | 12.39±1.91 | |  | 2.44 |

OIL, oil content; PRO, protein content; FIB, fiber content; OLE, oleic; LIO, linoleic; LIN, linolenic; PAL, palmitic; STR, stearic; Omega 3/6 linolenic to linoleic ratio; USAT/SAT, unsaturated to saturated ratio; OLE/USAT, oleic to unsaturated ratio; TTC, total tocopherol content; TPC, total phenolic content; ASP, aspartic; MET, methionine; ISOLEU, isoleucine; LEU, leucine; LYS, lysine; HIST, histidine, SDG, secoisolariciresinol diglucoside; SECO, secoisolariciresinol; MUC, mucilage.

| **Table S4.** Genotypic correlation coefficients between different traits in 108 flax genotypes (100 F6 families and 8 parental genotypes) during two years. | | | | | | | | | | | | | | | | | | | | | | |
| --- | --- | --- | --- | --- | --- | --- | --- | --- | --- | --- | --- | --- | --- | --- | --- | --- | --- | --- | --- | --- | --- | --- |
| Trait | Oil | PRO | FIB | OLE | LIE | LIN | PAL | STR | ASP | MET | ISOLE | LEU | LYS | HIS | Omega3/6 | USAT/SAT | OLE/USAT | TTC | TPC | SDG | SECO | MUC |
| Oil | **1** |  |  |  |  |  |  |  |  |  |  |  |  |  |  |  |  |  |  |  |  |  |
| PRO | 0.542** | **1** |  |  |  |  |  |  |  |  |  |  |  |  |  |  |  |  |  |  |  |  |
| FIB | 0.667 | 0.678** | **1** |  |  |  |  |  |  |  |  |  |  |  |  |  |  |  |  |  |  |  |
| OLE | 0.705** | 0.040 | -0.268 | **1** |  |  |  |  |  |  |  |  |  |  |  |  |  |  |  |  |  |  |
| LIE | 0.109 | -0.279 | -0.152 | -0.651** | **1** |  |  |  |  |  |  |  |  |  |  |  |  |  |  |  |  |  |
| LIN | 0.086 | -0.327 | 0.308 | -0.736** | -0.768** | **1** |  |  |  |  |  |  |  |  |  |  |  |  |  |  |  |  |
| PAL | 0.672** | 0.109 | 0.313 | 0.206 | -0.462* | -0.682** | **1** |  |  |  |  |  |  |  |  |  |  |  |  |  |  |  |
| STR | 0.682** | 0.209 | 0.293 | 0.256 | -0.542** | -0.632** | 1.000 | **1** |  |  |  |  |  |  |  |  |  |  |  |  |  |  |
| ASP | -0.091 | 0.822** | -0.280 | 0.317 | -0.156 | -0.258 | 0.909** | 0.279 | **1** |  |  |  |  |  |  |  |  |  |  |  |  |  |
| MET | 0.198 | 0.834** | -0.297 | 0.091 | -0.163 | -0.184 | 0.251 | -0.054 | 0.581** | **1** |  |  |  |  |  |  |  |  |  |  |  |  |
| ISOLE | 0.142 | 0.873** | -0.311 | 0.548** | -0.219 | -0.175 | 0.190 | 0.290 | 0.632** | 0.710** | **1** |  |  |  |  |  |  |  |  |  |  |  |
| LEU | 0.107 | 0.809** | -0.209 | 0.162 | -0.042 | 0.289 | 0.248 | 0.248 | 0.650** | 0.673** | 0.788** | **1** |  |  |  |  |  |  |  |  |  |  |
| LYS | 0.170 | 0.885** | -0.330 | -0.260 | 0.285 | -0.206 | -0.055 | -0.055 | 0.631** | 0.731** | 0.644** | 0.849** | **1** |  |  |  |  |  |  |  |  |  |
| HIS | 0.125 | 0.817** | -0.117 | 0.485* | -0.095 | 0.302 | -0.107 | -0.107 | 0.74** | 0.693** | 0.671** | 0.871** | 0.817** | **1** |  |  |  |  |  |  |  |  |
| Omega3/6 | -0.176 | -0.310 | 0.320 | 0.320 | -0.753** | 0.803** | -0.045 | -0.045 | 0.094 | 0.205 | 0.262 | 0.284 | 0.324 | 0.254 | **1** |  |  |  |  |  |  |  |
| USAT/SAT | 0.096 | 0.036 | -0.175 | 0.809** | 0.864** | 0.882** | -0.704** | -0.774** | -0.103 | -0.196 | 0.098 | 0.072 | -0.110 | -0.066 | -0.077 | **1** |  |  |  |  |  |  |
| OLE/USAT | 0.640** | -0.258 | 0.295 | 0.899** | -0.666** | -0.729** | 0.198 | 0.168 | 0.105 | -0.297 | 0.090 | 0.059 | 0.101 | 0.099 | 0.320 | -0.204 | **1** |  |  |  |  |  |
| TTC | 0.784** | 0.125 | 0.336 | -0.405 | 0.590** | 0.592** | -0.404* | -0.374 | 0.218 | 0.156 | 0.275 | 0.206 | 0.225 | 0.177 | -0.039 | 0.450* | 0.111 | **1** |  |  |  |  |
| TPC | 0.723** | 0.159 | 0.264 | -0.393 | 0.621** | 0.588* | -0.465* | -0.425* | 0.224 | 0.192 | 0.283 | 0.257 | 0.147 | 0.285 | 0.101 | 0.424* | 0.045 | 0.905*8 | **1** |  |  |  |
| SDG | 0.752** | 0.133 | 0.238 | -0.402* | 0.624** | 0.608** | -0.437* | -0.397 | 0.140 | 0.231 | 0.302 | 0.271 | 0.230 | 0.136 | -0.017 | -0.110 | -0.06 | 0.820** | 0.862** | **1** |  |  |
| SECO | 0.752** | 0.143 | 0.238 | -0.472* | 0.65** | 0.638** | -0.467* | -0.387 | 0.190 | 0.261 | 0.312 | 0.221 | 0.300 | 0.226 | -0.017 | -0.100 | -0.44 | 0.790** | 0.912** | 0.902** | **1** |  |
| MUC | 0.233 | -0.278 | 0.483* | 0.104 | -0.178 | 0.293 | 0.150 | 0.310 | -0.102 | 0.305 | -0.109 | -0.110 | -0.078 | -0.084 | 0.273 | 0.099 | 0.406 | -0.080 | 0.299 | 0.306 | 0.333 | **1** |

^*^P <0.05, ^**^P <0.01

OIL, oil content; PRO, protein content; FIB, fiber content; OLE, oleic; LIO, linoleic; LIN, linolenic; PAL, palmitic; STR, stearic; Omega 3/6 linolenic to linoleic ratio; USAT/SAT, unsaturated to saturated ratio; OLE/USAT, oleic to unsaturated ratio; TTC, total tocopherol content; TPC, total phenolic content; ASP, aspartic; MET, methionine; ISOLEU, isoleucine; LEU, leucine; LYS, lysine; HIST, histidine, SDG, secoisolariciresinol diglucoside; SECO, secoisolariciresinol; MUC, mucilage.
